# Characterizing the impact of intracutaneous dissemination on host responses during *Borrelia burgdorferi* infection

**DOI:** 10.3389/fimmu.2026.1850665

**Published:** 2026-06-05

**Authors:** Samantha Crane, Amira-Nuriya McKinney, Clayton Jarrett, Chad S. Clancy, Olof Rickard Nilsson, Kelly L. Hawley, Ashley M. Groshong

**Affiliations:** 1 Laboratory of Bacteriology, Rocky Mountain Laboratories, Division of Intramural Research, National Institute of Allergy and Infectious Diseases, National Institutes of Health, Hamilton, MT, United States; 2 Rocky Mountain Veterinary Branch, Rocky Mountain Laboratories, Division of Intramural Research, National Institute of Allergy and Infectious Diseases, National Institutes of Health, Hamilton, MT, United States; 3 Departments of Pediatrics, Medicine, and Immunology, University of Connecticut School of Medicine, Farmington, CT, United States; 4 Department of Research, Connecticut Children’s Research Institute, Hartford, CT, United States; 5 Divisions of Research, and Infectious Diseases and Immunology, Connecticut Children’s, Hartford, CT, United States

**Keywords:** *Borrelia burgdorferi*, dissemination, host response, humoral response, lyme disease

## Abstract

To investigate the unique dissemination phenotype of a mutant in OppA2 of *Borrelia burgdorferi* (*Bb*), one of the five encoded peptide binding proteins of the peptide transport system. Our previous study showed that loss of OppA2 abrogates hematogenous dissemination, restricting the bacteria to intracutaneous dissemination routes. Herein, we further define this unique dissemination phenotype and seek to understand the resultant dampening of the host serological response during infection. Methods: We utilized longitudinal infection models to evaluate dissemination, cytometric and histopathologic analysis, spatial immune profiling, ELISA, and host immune response transcriptomics. Results: Dissemination studies demonstrate that at 4 weeks post-inoculation (wpi), in addition to skin colonization, *oppA2tn* can be found in lymph nodes. By 8 wpi the *oppA2tn* mutant fully disseminates throughout the skin and by 20 wpi sporadic dissemination to distal organs. While immune cell populations do not show significant changes between wild-type and mutant infections, we see dramatic effects related to antibody responses, as well as host transcriptional responses during early infection. We further evaluated the roles of MyD88 signaling and the adaptive immune system (using MyD88 and SCID knockout mice, respectively) in limiting intracutaneous dissemination. We found that both immune components appear to control spirochete dissemination in the skin, as both mouse strains displayed faster skin colonization as well as distal site colonization. Conclusions: Colonization of lymph nodes at 4 week post-inoculation by the *oppA2tn* mutant strongly implies that eventual organ colonization arises from lymphatic dissemination, a phenomenon that has been difficult to study when paired with hematogenous dissemination. Additionally, the dampening of immune responses show that sequestration of the bacteria to skin during early infections results in integral changes in how the host both detects and responds to the spirochete. Together, these data shed light on the role hematogenous dissemination plays in generating a robust anti-*Bb* immunological response and suggests that distal sites of colonization are integral for eliciting typical serological responses and inflammatory profiles observed in wild type *Bb*-murine infections.

## 1 Introduction

The propagation of *Borrelia burgdorferi* (*Bb*), the causative agent of Lyme disease, in its natural enzootic cycle relies on the maintenance of infected reservoir species (1). As the spirochetes’ vector, *Ixodes scapularis*, does not have the capacity for transovarial transmission of the pathogen, larvae must acquire the bacteria during a bloodmeal from an infected animal (1). This dependency requires the reservoir species to maintain a robust dermal colonization of the bacteria as cohorts of ticks transition into their different life stages.

After deposition of the spirochete into the skin feeding site, systemic dissemination relies on a coordination of bacterial gene expression, targeted migration, and immune evasion (2–5). Host-adaptation relies on a major programmatic change in gene regulation which encompasses 1) shifting metabolic pathways to transition from the tick to the host, 2) repressing highly antigenic tick phase proteins, and 3) promoting surface protein expression that aids in dissemination and immune evasion within the host (3). As *Bb* migrates into the dermis, the bacteria is confronted with immune cells such as macrophages, dendritic cells, and neutrophils (6). Initial skin colonization is quickly followed by a transient spirochetemia which seeds spirochetes to distal organ sites, resulting in systemic dissemination by 2 weeks post-infection (wpi) (7, 8). Phagocytosed bacteria and *Bb-*associated PAMPs (pathogen-associated molecular patterns such as lipoproteins, peptidoglycan, RNA, DNA, etc.) result in immune signaling and cytokine/chemokine production (9). Sampling of *Bb*-associated PAMPs and antigen presentation to adaptive immune cells such as T and B cells result in pathogen specific responses, such as antibody production (9, 10). It is the robust immune response to *Bb* which drives much of the pathology related to Lyme disease.

Previously, we characterized a *Bb* transposon (*tn*) mutant deficient in oligopeptide binding protein 2 (*oppA2tn*) (11). OppA2 is one of five proteins that facilitate peptide binding and transport. Peptide transport is driven by the oligopeptide transport system, a complex ABC transporter encoded on the *Bb* chromosome and accessory plasmids (12). Peptide transport was shown to be critical for viability both *in vitro* and *in vivo*. Individually, OppA mutants have not shown defects in growth *in vitro*, likely due to enriched media and functional redundancy. However, we have demonstrated that *oppA1tn* and *oppA2tn* display unique *in vivo* phenotypes, suggesting unique roles for each during the enzootic cycle (11, 13). In the case of the *oppA2tn*, the spirochete was able to establish colonization within the skin (localized infection), though it was unable to hematogenously disseminate and spread to distal tissues (early-disseminated infection). Despite this hematogenous dissemination defect, when ticks were fed on *oppA2tn*-colonized mouse skin (*e.g.* inoculation site), ticks were able to both acquire and efficiently deliver the *oppA2tn* mutant during acquisition-transmission studies. Whether inoculation was performed by needle or tick, an extensive sampling of skin sites at 4 wpi showed that the mutant was able to proliferate and intracutaneously migrate through the skin, moving outward from the inoculation site with bacterial burdens similar to wild type (*wt*). At this timepoint, no mutant bacteria were cultured from distal sites such as the heart and joint tissues, whereas *wt* infections resulted in systemic dissemination by 2 wpi. Unexpectedly, infection with *oppA2tn* resulted in a minimal antibody response when compared with the *wt* infection, suggesting systemic dissemination is critical for mounting a robust immune response.

The role of intracutaneous migration during dissemination is not well defined due to the inability to uncouple the process of hematogenous dissemination from other routes. For clarity, we classify the ability to migrate into distal tissues as systemic dissemination regardless of route, hematogenous dissemination as the ability to disseminate via blood, and cutaneous dissemination as dissemination occurring via the skin excluding hematogenous means. In this study, we sought to further characterize cutaneous migration of the *oppA2tn* mutant to understand how this process impacts systemic dissemination in the absence of hematogenous dissemination. We also expanded our investigation into the host response to this cutaneous dissemination model, given the diminished antibody responses against the *oppA2tn* skin-resident spirochetes at 4wpi. Herein, we show that at 4 wpi on, *oppA2tn* spirochetes are also found in the lymph nodes (LNs), though at a lesser frequency than *wt*. Additionally, we characterized the longitudinal dynamics of *oppA2tn* infection. By 8 wpi, we find the mutant achieves complete intracutaneous dissemination though no distal tissues were colonized; by 20 wpi, we saw sporadic colonization of distal tissues. Utilizing the *oppA2tn* mutant as a model for cutaneous dissemination, we were able to demonstrate for the first time that both innate and adaptive immune responses play a role in dampening intracutaneous migration during infection. While *oppA2tn*-infected mice did eventually generate antibodies against classical *in vivo* antigens (FlaB, OspC, DbpA, VlsE) in immunocompetent mice, most antigen-specific responses were both reduced and intermittent. Histopathological assessments and cytometric analyses of immune cell populations in LNs and peripheral blood did not identify statistically significant shifts in immune cell populations for *oppA2tn*-infected mice. However, an evaluation of host transcriptional responses during early timepoints (5 dpi and 2 wpi) demonstrated significant immune dysregulation in *oppA2tn*-infected mice, specifically in the LNs. These data support an underappreciated role for cutaneous dissemination during infection and suggest that the evolutionary cost of effective and expedient dissemination rendered via the hematogenous route is a quick and robust immune response from the host.

## 2 Methods and materials

### 2.1 Bacterial strains and culture conditions

*Escherichia coli* strains (**Supplementary Table 1**) were grown in Luria-Bertani (LB) broth or on LB plates with appropriate antibiotics (ampicillin [Amp; 100 μg/ml] or kanamycin [Kan; 50 μg/ml]) at 37°C. *Borrelia burgdorferi* strains used in this study (**Supplementary Table 1**) are the wild-type strain (14) B31 5A18 NP1 (*wt*) and the *oppA2* transposon mutant (11) in strain B31 5A18 NP1 (*oppA2tn*). *Bb* strains were cultured in modified Barbour-Stoenner-Kelly II (BSK-II) (15) medium supplemented with 6% rabbit serum and relevant antibiotics (kanamycin [Kan; 400 μg/ml] or gentamicin [Gent; 50 μg/ml]) at 37°C in a CO_2_ incubator (5%) unless otherwise noted.

### 2.2 Ethics statement

All animal work was conducted according to the guidelines of the National Institutes of Health, *Public Health Service Policy on Humane Care and Use of Laboratory Animals* (16), and the United States Institute of Laboratory Animal Resources and National Research Council, *Guide for the Care and Use of Laboratory Animals* (17). All protocols were approved by the Rocky Mountain Laboratories, NIAID, NIH Animal Care and Use Committee. The Rocky Mountain Laboratories are accredited by the Association for Assessment and Accreditation of Laboratory Animal Care (AAALAC) International.

### 2.3 Infections

All animal infections were performed with 4-6w old mice. Immunocompetent mouse studies utilized C3H/HeJ mice (Jackson Laboratory). Immunocompromised mouse studies used either C3H *MyD88*^-/-^ (18), a kind gift from Linda Bockenstedt (Yale University School of Medicine, New Haven, Connecticut, USA) or C3H *SCID* (C3SnSmn.Cg-*Prkdc^scid^*/J) mice (Jackson Laboratory). *Wt* and *oppA2tn* strains were used to intradermally infected mice in a central dorsal location. At 5d, 9d, 2w, 4w, and 8w, mice were sacrificed for tissue collection for downstream culturing and assays. For long term infections (20w), sera was collected from mice every two weeks. Tissues (ear, inoculation site, tibiotarsal joint, heart, bladder, proper axillary (PALN), Accessory axillary (AALN), and sub-iliac (SiLN) lymph nodes (19)[**Supplementary Figure 1D**]) were either cultured in modified BSK-II supplemented with *Borrelia* antibiotic mixture (BAM; 0.05 mg/ml sulfamethoxazole, 0.02 mg/ml phosphomycin, 0.05 mg/ml rifampicin, 0.01 mg/ml trimethoprim, and 2.5 µg/ml amphotericin B), preserved in RNAlater (Invitrogen) for RNA analysis, or fixed in 10% formalin for histopathological analysis.

### 2.4 Immunoblots

Immunoblots were performed as previously described (11). Antibody responses were evaluated by probing *Bb* whole cell lysates with sera from infected mice (1:1,000) and a horseradish peroxidase-conjugated goat anti-mouse secondary antibody (Southern Biotechnology Associates, Birmingham, AL) at 1:20,000. Immunoblots were developed using the SuperSignal West Pico chemiluminescence substrate (Pierce, Rockford, IL).

### 2.5 Histopathology

Sections of haired skin, lymph node, heart and whole hind limb (tibiofemoral joint) from 5 dpi, 4 wpi, and 8 wpi timepoints were evaluated for histopathologic changes. Tissues were preserved in 10% neutral buffered formalin for a minimum period of seven days. Hind limbs were decalcified as needed using standard protocols in a 10% solution of ethylenediaminetetraacetic acid (EDTA). Tissues were placed in cassettes and processed with a Sakura VIP-6 Tissue Tek on a 12-hour automated schedule using a graded series of ethanol, xylene and PureAffin. Prior to hematoxylin and eosin (H&E) staining, embedded tissues were sectioned at 5 µm and dried overnight at 42°C. All tissues were analyzed by a blinded, board certified veterinary anatomic pathologist. Tissues were graded on a scale of 0 (no lesion) to 4 (severe lesion), visually represented by the histopathological scoring matrix in **Supplementary Figure 2A**. Representative images of histopathologic lesion scores were captured on an Olympus DP80 camera mounted to an Olympus BX51 microscope using cellSens Dimension software (Olympus) at 100x magnification.

### 2.6 Meningeal immunofluorescence assay

Mice were perfused with heparin saline (100 U/ml) and skull cap with meninges attached were excised and fixed in 4% paraformaldehyde overnight at 4°C. The skull cap was rinsed in phosphate-buffered saline (PBS) and meninges were gently removed. Meninges were placed in individual wells in a 24-well plate, where all staining was performed using gentle shaking. Meninges were washed twice with PBS for 5m, blocked in blocking buffer (2% normal donkey serum, 1% bovine serum albumin, 0.1% Triton X-100, 0.05% Tween 20 in PBS) for 1h at room temperature (RT) and incubated with primary antibody diluted in blocking buffer (rabbit anti-CD31, 1:1000 dilution, Abcam; goat anti-Borrelia, 1:400 dilution, SeraCare) overnight at 4°C. Following two PBS washes for 10m each, meninges were incubated with secondary antibody (donkey anti-rabbit AF568, 1:400 dilution, Thermo; donkey anti-goat AF488, 1:400 dilution, Thermo) diluted in blocking buffer for 1h at RT. After two PBS washes for 10m each, meninges were blocked for two hours at RT with a second blocking buffer containing 2% normal rabbit serum in place of normal donkey serum to avoid cross-binding of the second primary conjugated antibody. Meninges were then incubated with the second conjugated primary antibody (rabbit anti-IBA-1 635, 1:400 dilution, Wako) diluted in the blocking buffer using rabbit serum overnight at 4°C. The following day, samples were washed twice for 10m each and mounted on microscopy slides using Prolong glass with NucBlue. Slides were allowed to cure at RT and imaged using a Zeiss LSM710 confocal microscope.

### 2.7 CBC panels

Hematology was analyzed on whole blood collected in EDTA micro sample tube (Microtube 1.3 ml K3E, Sarstedt) using a ProCyte DX (IDEXX Laboratories). The parameters analyzed included red blood cells, hemoglobin, hematocrit, mean corpuscular volume, mean corpuscular hemoglobin, mean corpuscular hemoglobin concentration, red cell distribution width, platelets, mean platelet volume, white blood cells, neutrophil count, lymphocyte count, monocyte count, eosinophil count, reticulocyte and basophil count.

### 2.8 Flow cytometry

LNs (PALN, AALN, SiLN) were collected from mice on one side at 5 dpi and 8 wpi. Pooled LNs were macerated through a 100 μm cell strainer and rinsed using 12ml BenchStable™ DMEM (Gibco) supplemented with 5% fetal bovine serum (FBS, Gibco) to retrieve single cell suspension. Throughout processing, cells were centrifuged at 500 × *g* for 5m. Cells were pelleted for 5m and then treated with 1X eBioscience RBC lysis buffer (Invitrogen) for 5m to lyse red blood cells. LN cells were pelleted for 5m and resuspended in 1ml PBS for enumeration using a Scepter 3.0 cell counter (MilliporeSigma). Cells (1.5x10^5^ total) were resuspended in diluted Zombie Yellow™ fixable cell viability dye (1:1,000 in PBS; Biolegend) and incubated at RT protected from light for 30m to stain non-viable cells. Cells were washed in 100 μl PBS and incubated in 50 μl TruStain FcX PLUS working buffer (1:100 dilution in 3% FBS in PBS, Biolegend) for 10m on ice protected from light to block Fc receptors. Cells were then washed with 100 µl 3% FBS in PBS and stained with the following antibodies for 30m at 4°C to stain cell surface markers: B220/CD45R-BUV615 (1:80 dilution; BD ABiosciences Clone RA3-6B2), CD3-BUV661 (1:80 dilution; BD Biosciences Clone 17A2), CD11b-BB515 (1:80 dilution; BD Biosciences Clone M1/70), CD11c-BUV563 (1:80 dilution; BD Biosciences Clone N418), CD19-AF700 (1:200 dilution; Biolegend Clone 6D5), CD45-PerCP Cy5.5 (1:80 dilution; Biolegend Clone D3F8Q), F4/80-BV650 (1:40 dilution; Biolegend Clone BM8), TCRγδ-BV605 (1:80 dilution; Biolegend Clone GL3), CD4-BUV395 (1:80 dilution; BD Biosciences Clone GK1.5), CD8α-BV510 (1:50 dilution; Biolegend Clone 53-6.7). Cells were washed in 100 µl 3% FBS in PBS, then resuspended in 100 µl Cytofix Fixation Buffer (BD) and incubated on ice for 45m for fixation. Cells were washed in 3% FBS in PBS before final cell resuspension in 3% FBS in PBS. The following day, cells were analyzed by flow cytometry on BD FACS Symphony A5 and BD FACSDiva. Flow cytometry data were evaluated and visualized using FlowJo software (version 10.8.1). Gating strategies can be found in **Supplementary Figure 3B**: B cells (G3: CD45^+^, CD3^-^, B220^+^, CD19^+^), CD4^+^ T cells (G1: CD45^+^, CD3^+^, TCRγδ^-^, CD4^+^), CD8^+^ T cells (G2: CD45^+^, CD3^+^, TCRγδ^-^, CD8^+^), macrophages (G4: CD45^+^, CD3^-^, F4/80^+^, CD11b^+^), and dendritic cells (G5: CD45^+^, CD3-, F4/80^lo/-^, CD11c^+^). Gating strategies were determined by staining patterns observed using fluorescence minus one (FMO) samples.

### 2.9 Protein expression and purification

The coding regions for FlaB, OspC, OspA, lp6.6, and GlpA were codon optimized for expression in *E. coli* and primers (**Supplementary Table 2**) were used to amplify gene fragments using Cloneamp HiFi Premix (Takara Bio). Gel purified fragments were cloned into pET28a (Novagen) and linearized with NdeI/XhoI using the InFusion HD cloning kit (Takara Bio) per manufacturer protocol. Cloning reactions were transformed into *E. coli* Stellar cells and positive transformants were selected with Kan. Individual clones were screened by PCR and confirmed by Sanger sequencing. pProEX HTa::DbpA (20) was kindly gifted by Jon Blevins.

Expression plasmids for FlaB, OspC, and DbpA were transformed into *E. coli* BL21 (DE3) expression strain (Invitrogen) and protein expression was induced with 1mM IPTG for 3h at 37°C. Expression plasmids for Lp6.6, OspA, and GlpA were transformed into *E. coli* C41 (DE3) expression strain (Sigma-Aldrich) and protein expression was induced via standard autoinduction methods as previously described (21). Proteins were purified and subjected to size exclusion chromatography as previously described (22), with the exception of FlaB, where 8 M urea was added during the purification process. Final buffers for proteins consisted of 10–20 mM phosphate/500 mM NaCl with a final concentration of 4 M urea for FlaB.

### 2.10 ELISA

Serum from *wt-* and *oppA2tn-*infected mice was collected every 2w for 20w. Concentrations of whole *Bb*, FlaB, OspC, DbpA, lp6.6, OspA, GlpA, or C6 peptide from VlsE (Alpha Diagnostics, San Antonio, TX) reactive IgG were determined using quantitative ELISA. Briefly, clear 96-well flat bottom Immuno plates (Thermo) were coated with *Bb* whole cell lysate (3 μg per well) or purified protein (4 ng per well) overnight at 4°C. Plates were then blocked (4% w/v Bacto™ TC Lactalbumin Hydrolysate, 15% normal goat serum, 0.5% Tween20, and 0.05% w/v sodium azide) for 1h at room temperature. Plates were subsequently washed with PBS/0.1% Tween20. Plates were then incubated with serial dilutions of serum for 1h at room temperature. After three washes, rabbit α-mouse IgG (H+L) HRP conjugated antibody (Invitrogen) was applied to plates in blocking solution without sodium azide at 1:10,000 and incubated for 1h at room temperature. Plates were washed three times and developed with 100 μl 1Step Ultra TMB (Thermo) for 15m at room temperature. 100 μl of ELISA Stop Solution (Invitrogen) was added to stop HRP reaction and OD_450_ was read using a ACTGene AgileReader plate reader. IgG quantification was performed using standard curves of purified mouse IgG polyclonal antibody (Invitrogen).

### 2.11 MACSima spatial profiling

Skin and LN samples were collected from mice at 5d, 4w, and 8 wpi and fixed in 10% formalin for 24 hours prior to paraffin embedding and prepared as described above. Prior to staining, antigen retrieval was performed on mounted tissues using a Roche Discovery Ultra (Roche) instrument using Medium Antigen Retrieval protocol. Briefly, slides were deparaffinized by heating slides to 69°C for 27 minutes, followed by incubation in Roche CC1 (Roche) buffer at 100°C for 56 minutes. Slides were then washed with Dawn dish soap (Proctor and Gamble) and stored in 1X MACSima Running Buffer (Miltenyi Biotec). Slides were mounted using MACSwell One or MACSwell Four Imaging Frames, and DAPI stained for 20m prior to analysis. Samples were processed on MACSima Imagining Platform (Miltenyi Biotec) using manufacturer’s protocols. Skin samples were stained using the following antibody panel with 1:50 dilution unless otherwise indicated: CD3e-AF555 (Cell Signaling Clone E5T2B), CD4-AF647 (1:100 dilution, Abcam Clone EPR19514), CD45R/B220-FITC (Miltenyi Biotec Clone REA755), CD68-AF488 (Cell Signaling Clone E307V), CD11b-AF555 (Cell Signaling Clone E4K8C), Ly6G-APC (Miltenyi Biotec Clone REA526), MPO-AF647 (Cell Signaling Clone E2Z8J), CD45-AF647 (Cell Signaling Clone D3F8Q), Vimentin-AF488 (Cell Signaling Clone D21H3), Pan-cytokeratin-AF488 (Invitrogen Clone AE1/AE3), Collagen I-AF647 (Abcam Clone EPR24331-53), EpCAM-AF488 (Abcam Clone EPR20532-222), in house conjugated Langerin-FITC (Abcam Clone EPR12685-12), CD31-AF647 (Abcam Clone EPR17259), and LYVE1-APC (Abcam Clone EPR21771). LNs were stained using the following antibody panel using the same antibody clones as in skin with 1:50 dilution unless otherwise indicated: CD3e-AF555 (Cell Signaling), CD4-AF647 (1:100 dilution, Abcam), CD45R/B220-FITC (Miltenyi Biotec), CD68-AF488 (Cell Signaling), CD11b-AF555 (Cell Signaling), Ly6G-APC (Miltenyi Biotec), MPO-AF647 (Cell Signaling), CD45-AF647 (Cell Signaling), Vimentin-AF488 (Cell Signaling), Collagen I-AF647 (Abcam), CD31-AF647 (Abcam), and LYVE1-APC (Abcam). Following MACSima processing, preprocessed image files were analyzed using MACS iQ View software (Miltenyi Biotec) and one field per mouse per tissue was observed. The following populations were identified by the following staining patterns: T cells (CD45^+^ CD3^+^), CD4^+^ T cells (CD45+ CD3+ CD4+), B cells (CD45R^+^), neutrophils (CD45^+^ CD11b^+^ Ly6G^+^), macrophages (CD45^+^ CD68^+^), Langerhans cells (CD45^+^ Langerin^+^), classical dendritic cells and monocytes (CD45^+^ CD11b^+^), blood vessels (CD31^+^), and lymphatic vessels (LYVE1^+^).

### 2.12 RNA purification and transcriptome analysis

Tissues were collected at 9d, 2w, 4w, and 8 wpi. Tissues were then homogenized with the TissueLyser LT (Qiagen), and RNA was isolated using the Direct-zol RNA Miniprep Kit (Zymo Research) using manufacturer’s directions. For Nanostring nCounter analysis, the nCounter Sprint Profiler system was used. RNA quality was verified using the Agilent 2200 TapeStation prior to use in nCounter analysis. For each sample, 50 ng of RNA was used for analysis using the murine host response 775 gene expression panel. Data from nCounter Sprint runs was analyzed and normalized in nSolver 4.0 and visualized using GraphPad Prism 10.4.1. PCA plots were visualized using R Studio (23–25).

### 2.13 Statistics

Statistical comparisons between *wt* and *oppA2tn* were performed using 2-way ANOVA. All Nanostring data was evaluated used the nSolver 4.0 software suite, genes were considered differentially expressed if FC > 2 and *p <*0.05.

## 3 Results

### 3.1 Systemic dissemination of *Bb* via lymphatic routes is significantly impaired in an intracutaneous dissemination model

**Figure 1:**
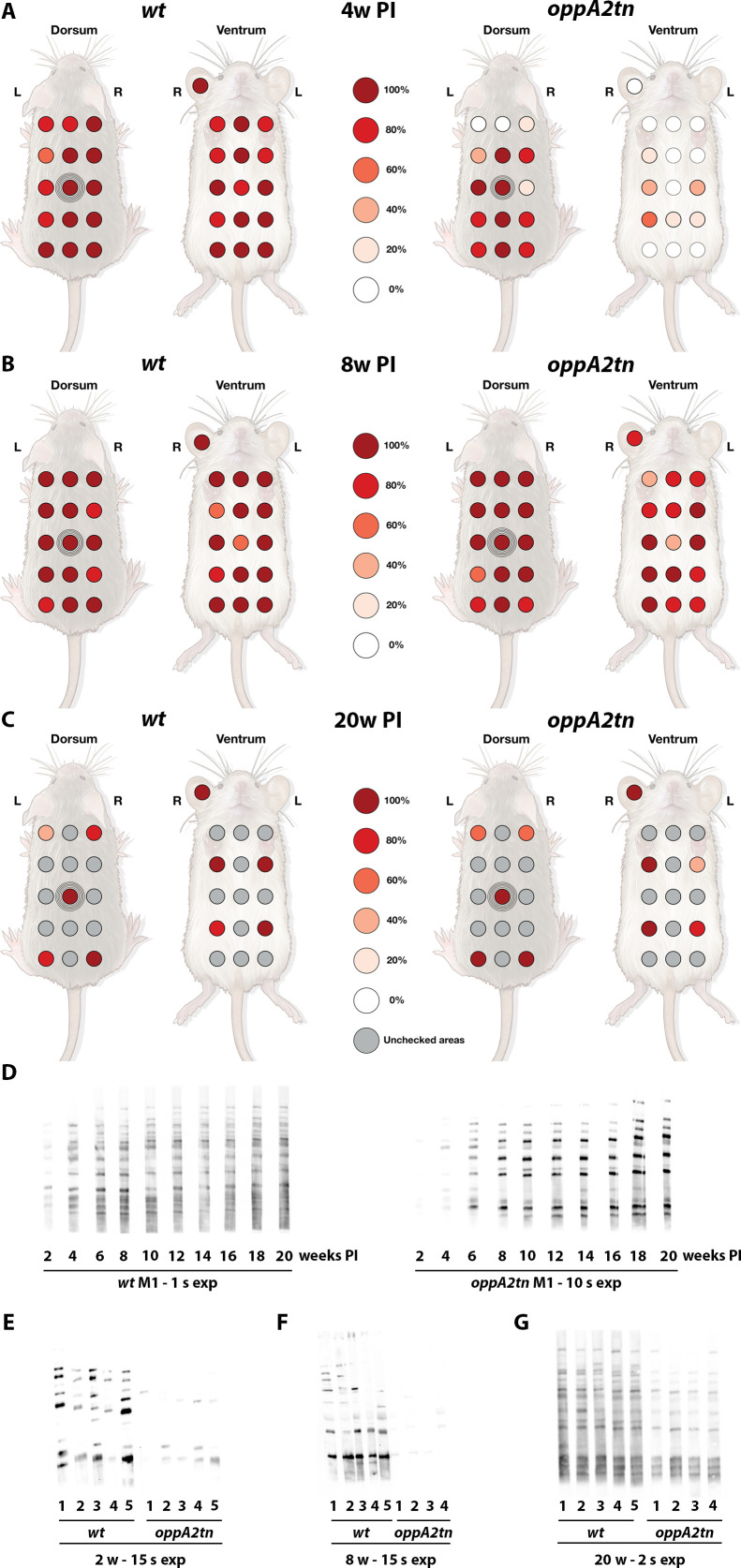
Longitudinal dissemination and serological response of the *oppA2tn* mutant. **(A-C)** Cartoon distribution of skin sites on the dorsum and ventrum of each mouse sampled during infection and percent of sites that cultured positive at **(A)** 4 wpi as described in Groshong et al. (11), **(B)** 8 wpi, and **(C)** 20 wpi. Tabulated results can be found in **Supplementary Table 3** and skin sampling maps in **Supplementary Figures 1A, B**. **(D–G)** Immunoblots of infected sera against whole cell *Bb* lysates. **(D)** Representative longitudinal immunoblot of single mice. Comparative immunoblots of *wt* and *oppA2tn* infected sera at **(E)** 2 wpi, **(F)** 8 iw, and **(G)** 20 wpi. Exposure times for each blot are included to aid in comparison.

Our previous study characterized infectivity of the *oppA2tn* mutant at 4 wpi (11). We showed that infection with the *oppA2tn* mutant resulted in intracutaneous dissemination which spread outward from the inoculation site but did not fully migrate throughout the skin at this timepoint (**Figure 1A**; **Supplementary Figure 1A**; **Supplementary Table 3**) (11). Notably, blood from the mice was culture negative during the acute phase of spirochetemia (3–7 dpi), and distal tissues were all culture negative at 4 wpi. qPCR of skin samples demonstrated bacterial burdens similar to *wt*, which showed that colonization within the skin was not impaired. Conversely, as early as 2 wpi, *wt* spirochetes can be cultured from all skin sites and distal tissues (commonly joints, bladder, and heart) (8). These data suggest that *oppA2tn* is unable to hematogenously disseminate, and thus, the *oppA2tn* infection model provides a valuable tool to further evaluate intracutaneous dissemination within the host. As needle- and nymph-inoculation of the *oppA2tn* mutant resulted in similar phenotypes (11), herein, we used needle-inoculation to bypass the lengthy process for generation of infected nymphs and the need for targeted feeding sites during acquisition.

*Bb* can be cultured from infected LN as early as 7d post-infection (dpi) and the presence of spirochetes in the LN results in significant lymphoadenopathy (26). Given that skin resident spirochetes may be able to localize to LNs with draining interstitial fluid, we assessed colonization of individual LNs (proper axillary LN [PALN], accessory axillary LN [AALN], and sub-iliac LN [SiLN]) at 4 wpi (**Table 1**; **Supplementary Figure 1D**). We found that some LNs were colonized in *oppA2tn*-infected mice. However, we observed fewer infected LNs than with the *wt*, suggesting the restriction to the skin compartment slows overall migration and colonization of these sites.

**Table 1:** Positive lymph node cultures for immunocompetent mice at four weeks post-inoculation.

4 w PI	Lymph node culture	
Tissue	*wt*	*oppA2tn*
Right PALN	5/5	2/5
Left PALN	5/5	1/5^†^
Right AALN	5/5	2/5
Left AALN	5/5	1/5^†^
Right SiLN	5/5	0/5^†^
Left SiLN	5/5	2/5
Total nodes	30/30	8/30

*LN locations are shown in **Supplementary Figure 1D**.

† *p* value <0.05 against *wt* using Fisher’s Exact test

To determine whether the *oppA2* mutant may be able to utilize lymphatic dissemination to invade distal tissues, we extended our studies to 8 and 20 wpi. At 8 wpi, we replicated the 30-point tissue culture sampling procedure previously used (**Supplementary Figure 1A**) (11). At 8 wpi, nearly all skin sites were culture positive for both *wt* and the *oppA2tn* mutant, showing that the mutant can fully disseminate within the skin given enough time (**Figure 1B**; **Table 2**; **Supplementary Table 3**). Distal tissues, however, were only culture positive for mice infected with *wt*. At 20 wpi, we used an abbreviated skin sampling procedure (**Supplementary Figure 1B**) and both strains showed similar infection incidence of skin sites, and *oppA2tn* sporadically colonized distal tissues, most notably in the tibiotarsal joints (**Figure 1C**; **Table 3**; **Supplementary Table 3**). These data suggest that spirochetes can utilize lymphatic dissemination to reach distal tissues, though the process is inefficient compared to hematogenous dissemination.

**Table 2:** Positive tissue cultures for immunocompetent mice at eight weeks post-inoculation.

8 w PI	Tissue culture	
Tissue	*wt*	*oppA2tn*
Ear*	5/5	4/5
Inoculation site*	5/5	5/5
Dorsal skin sites*	67/70	66/70
Ventral skin sites*	70/75	64/75
Tibiotarsal joint	5/5	0/5^†^
Bladder	5/5	0/5^†^
Heart	5/5	0/5^†^
Total tissues	163/170	140/170^†^
Total mice	5/5	5/5

*See **Figure 1B**, **Supplementary Figure 1A** and **Supplementary Table 3** for specific skin site related culture data.

† *p* value <0.05 against *wt* using Fisher’s Exact test

**Table 3:** Positive tissue cultures for immunocompetent mice at twenty weeks post-inoculation.

20 w PI	Experiment 1		Experiment 2	
Tissue	*wt*	*oppA2tn*	*wt*	*oppA2tn*
Ear*	5/5	3/4	5/5	5/5
Inoculation site*	5/5	4/4	5/5	5/5
Dorsal skin sites*	13/20	11/16	16/20	16/20
Ventral skin sites*	18/20	12/16	19/20	12/20
Tibiotarsal joint	5/5	2/4	5/5	4/4
Bladder	5/5	0/4^†^	5/5	1/5^†^
Heart	5/5	0/4^†^	5/5	0/5^†^
Total tissues	56/65	32/52^†^	60/65	43/65^†^
Total mice	5/5	4/4	5/5	5/5

*See **Figure 1C**, **Supplementary Figure 1B**, and **Supplementary Table 3** for specific skin site related culture data.

† *p* value <0.05 against *wt* using Fisher’s Exact test

Our previous 4w study also observed that mice infected with the *oppA2tn* mutant had a markedly reduced serological response toward *Bb* as assessed by immunoblot against whole cell lysates (11). To evaluate *Bb-*specific antibody generation longitudinally, we evaluated seroreactivity via immunoblot at 2w intervals during infection (**Figures 1D–G**, **Supplementary Figure 1C**). We observed an increase in antibody response for *oppA2tn*-infected mice, specifically within the first 8w of infection (**Figure 1D**). However, when infection cohorts are compared within a single timepoint, *oppA2tn*-infected mice showed significantly reduced antibody response compared to *wt* infections (**Figures 1E–G**, **Supplementary Figure 1C**). Additionally, immunoblots with sera from mice infected with *wt* identify a greater number of seroreactive proteins as compared with *oppA2tn*-infected sera immunoblots.

### 3.2 Both the innate and adaptive immune responses curtail intracutaneous migration

**Figure 2:**
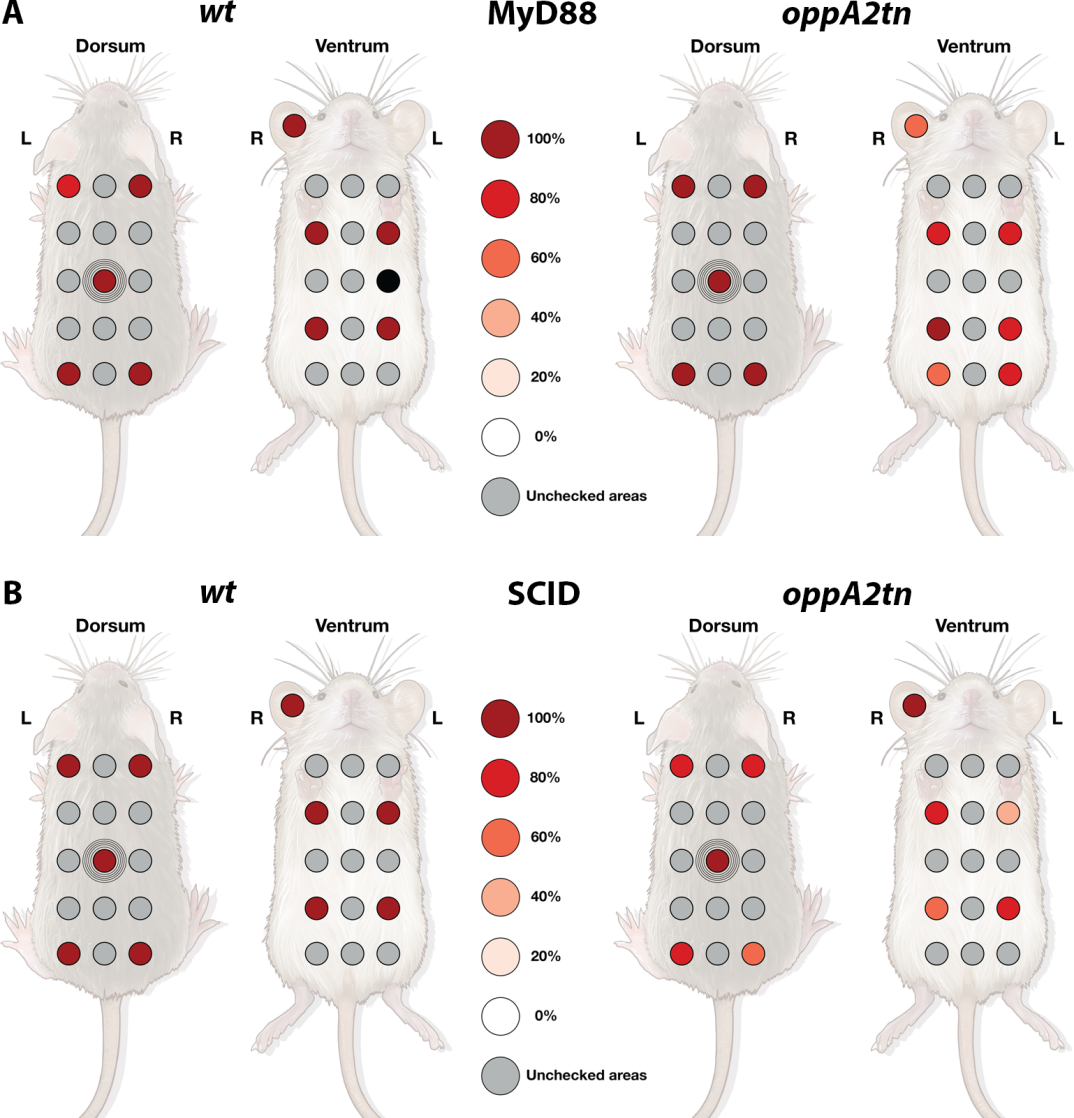
Dissemination of the *oppA2tn* mutant in immunocompromised mice. **(A, B)** Cartoon distribution of skin sites on the dorsum and ventrum of each mouse sampled during infection and percent of sites that cultured positive at 4 wpi for **(A)** C3H/MyD88 and **(B)** C3H/SCID mice. Tabulated results can be found in **Supplementary Table 3** and skin sampling maps in **Supplementary Figure 1B**.

Given that the *oppA2tn* infection model has provided unique insight into intracutaneous dissemination, we sought to understand the role of immune responses in controlling dissemination within the skin and via the lymphatic system. To this end, we evaluated the infectivity phenotype of the *oppA2tn* mutant in C3H/*MyD88* mice and C3H/*SCID* mice at 4 wpi, to evaluate the innate and adaptive contributions to control of intracutaneous dissemination, respectively We chose 4 wpi, a timepoint where we saw limited skin dissemination (**Figure 1A**), lymphatic invasion (**Table 1**), and no distal tissue colonization in immunocompetent mice (11), as we hypothesized if either arm of the immune system was involved, this timepoint would provide the greatest discrepancies between the immunocompetent and immunodeficient mouse models. Mice were inoculated in two independent experiments for each mouse model and tissue culture positivity was assessed for our abbreviated skin model (**Supplementary Figure 1B**) and distal tissues at 4 wpi (**Figure 2**; **Table 4**; **Supplementary Table 3**). Both the MyD88 and the SCID infections demonstrated increased colonization of skin sites distal from the inoculation site (*e.g.* ventrum) as compared with immunocompetent mice at 4 wpi, showing intracutaneous dissemination was more efficient in the absence of each arm of the immune response. Additionally, we found that distal tissues were culture positive at this timepoint, though not as consistently as *wt*-infected mice. While heart tissues were not culture positive for *oppA2tn*-infected immunocompetent mice even at 20 wpi (**Table 3**), we found that some heart tissues were culture positive in immunodeficient mice, suggesting greater systemic dissemination in the absence of immune controls. These data confirm that both the innate and adaptive immune responses influence the efficiency of cutaneous dissemination and the propensity for these *oppA2tn* spirochetes to reach distal organs.

**Table 4:** Positive tissue cultures for immunocompromised mice at four weeks post-inoculation.

C3H/MyD88–4 w	Experiment 1		Experiment 2	
Tissue	*wt*	*oppA2tn*	*wt*	*oppA2tn*
Ear*	5/5	5/5	5/5	1/5^†^
Inoculation site*	5/5	5/5	5/5	5/5
Dorsal skin sites*	20/20	20/20	17/20	20/40
Ventral skin sites*	20/20	20/20	20/20	7/20^†^
PALN	2/5	5/5	ND	ND
AALN	1/5	5/5	ND	ND
SiLN	1/5	5/5	ND	ND
Tibiotarsal joint	5/5	5/5	5/5	1/5**^†^
Bladder	N/A	N/A	5/5	1/5**^†^
Heart	4/5	1/5^†^	5/5	1/5**^†^
Total tissues	64/75	71/75	62/65	36/65^†^
Total mice	5/5	5/5	5/5	5/5
C3H/SCID 4 w	Experiment 1		Experiment 2	
Tissue	*wt*	*oppA2tn*	*wt*	*oppA2tn*
Ear*	5/5	4/5	5/5	5/5
Inoculation site*	5/5	5/5	5/5	5/5
Dorsal skin sites*	20/20	12/20^†^	20/20	15/20^†^
Ventral skin sites*	20/20	10/20^†^	20/20	15/20^†^
PALN	3/3	2/3	ND	ND
AALN	4/4	0/1	ND	ND
SiLN	5/5	4/5	ND	ND
Tibiotarsal joint	5/5	5/5	5/5	5/5
Bladder	ND	ND	5/5	5/5
Heart	5/5	1/5^†^	5/5	5/5
Total tissues	72/72	43/69^†^	65/65	55/65^†^
Total mice	5/5	5/5	5/5	5/5

*See **Figure 2**, **Supplementary Figure 1B**, and **Supplementary Table 3** for specific skin site related culture data, ND – not determined.

** Same mouse was positive for these tissue cultures

† *p* value <0.05 against *wt* using Fisher’s Exact test

### 3.3 Restriction to intracutaneous migration does not change histopathology or immune cell populations in infected tissues

**Figure 3:**
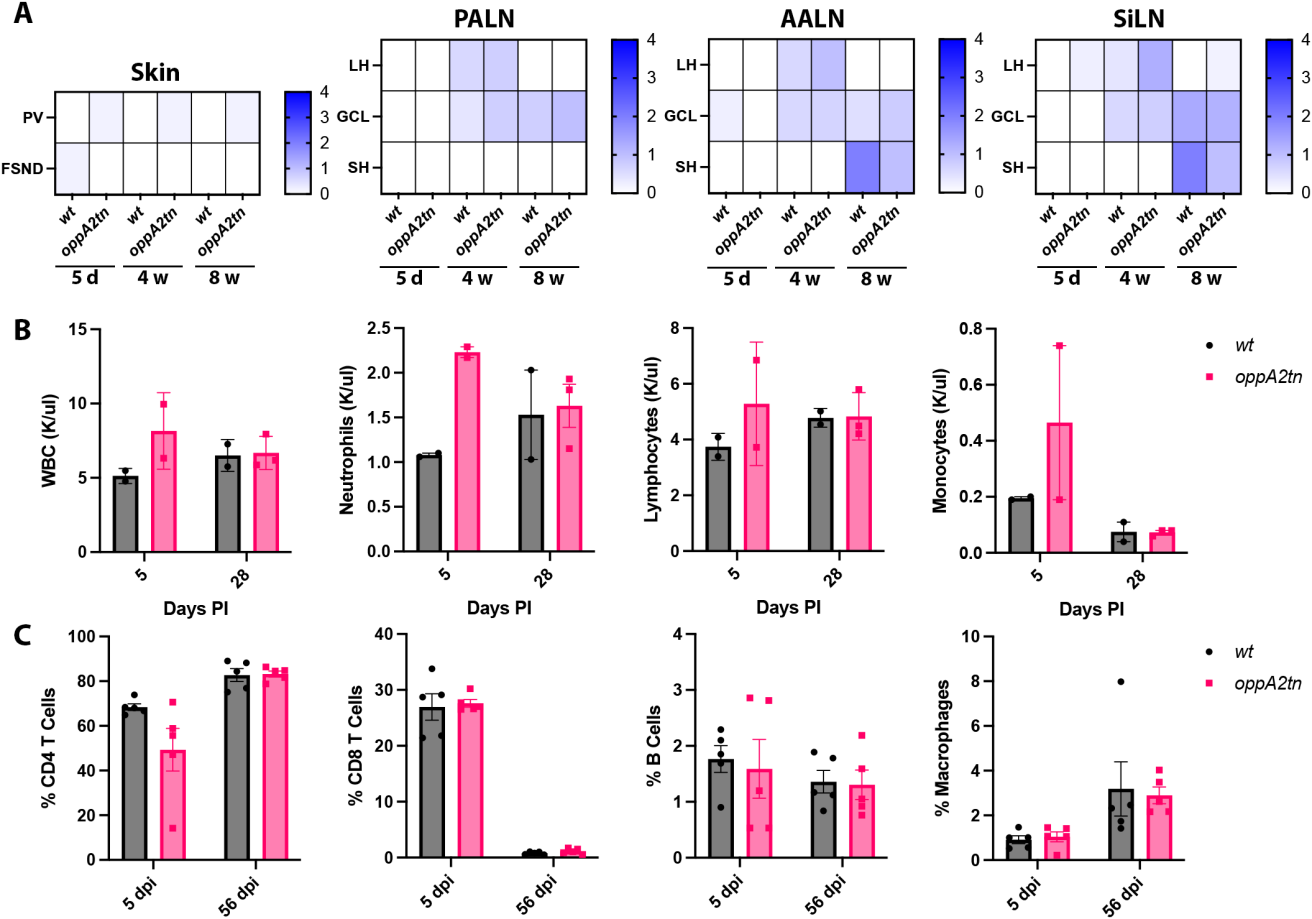
Tissue and cellular characterization of *wt* and *oppA2tn* infections. **(A)** Heatmap of histopathological scores for skin and lymph nodes. PV = perivasculitis, FSND = focal superficial necrotizing dermatitis, LH = lymphoid hyperplasia, GCL = germinal center lympholysis, SH = sinus histiocytosis. **(B)** CBC results for white blood cells (WBC), neutrophils, lymphocytes, and monocytes at 5 dpi and 4 wpi (28 d). **(C)** Flow cytometry of LNs at 5 dpi and 4 wpi (28 d) for CD4^+^ T cells and CD8^+^ T cells (CD4^+^ and CD8^+^ subpopulations of live CD45^+^/TCRγδ^-^/CD3^+^ cells, respectively), B cells and macrophages (B220^+^/CD19^+^ and F4/80^+^ subpopulations of live CD45^+^/CD3^-^ cells, respectively). Gating strategies for cell populations can be found in **Supplementary Figure 3B**. Points represent individual mice, bars represent mean, and error bars represent SEM; no statistical significance was found for panels B-C using 2-way ANOVA.

Despite both innate and adaptive immune involvement in controlling *oppA2tn* cutaneous dissemination, we noted a significant impairment in serological responses in the *oppA2tn* infection model. To further characterize the impact of colonization with *oppA2tn*, we performed histopathological analyses on skin (inoculation site), lymph nodes, at 5 dpi, 4 wpi, and 8 wpi and heart and tibiofemoral joint at 4 wpi. As expected, significant histopathologic differences were observed between *wt* and *oppA2tn* in the heart and tibiofemoral joint (**Supplementary Figure 2B**). Characteristics of inflammation in the heart were scored at moderate severity in *wt*-infected mice, with only minimal scoring in *oppA2tn*-infected mice. Similarly, only the tibiofemoral joint of *wt*-infected mice showed moderate scoring. These differences are consistent with systemic dissemination of *wt* at 4w, when *oppA2tn* is not present in these tissues. Histopathologic evaluation of tissues where both *wt* and *oppA2tn* had established colonization (skin and LNs) revealed no difference between the two strains (**Figure 3A**). We observed little to no incidence of perivasculitis and focal superficial necrotizing dermatitis in inoculation site skin of *wt-* and *oppA2tn*-infected mice over the course of infection. Alternatively, we observed similar incidence of lymphoid hyperplasia, germinal center lympholysis, and sinus histiocytosis in PALN, AALN, and SiLN pathology. However, sinus histocytosis scores were elevated, albeit still mild, in AALN and SiLN at 8 wpi in *wt*-infected mice compared to *oppA2tn*-infected mice, indicating increased antigen presenting cell trafficking to the subcapsular or medullary sinus region of theses LNs. Notably, PALN did not display scorable sinus histocytosis in either strain, this may suggest that groups of LNs are somewhat asynchronous in histolopathological development. This is not wholly unexpected as we inoculated in the central dorsal region instead of a site where we could specifically collect the draining LNs of the inoculation site. Together, these data indicate *Bb* hematogenous dissemination to distal tissues is required for increased inflammation and pathology, while infected tissues appear similarly impacted by both *wt* and *oppA2* infection.

Additionally, we evaluated the impact of *Bb* hematogenous dissemination on mouse meninges (**Supplementary Figure 2C**). Previous studies have shown *Bb* burdens peak in the meninges by 7 dpi resulting in increased inflammation and perivascular hemorrhage (27). We were particularly interested in this site as it has been shown that *Bb* can be found in cerebrospinal fluid (CSF) within the same infection window as the blood, though it is unclear how invasion of the blood, CSF, and lymph may coordinate systemic dissemination (27). While we were unable to detect *wt* or *oppA2tn Bb* in the meninges at 4 wpi, there was an obvious increase in IBA-1 staining and striation, indicative of increased macrophage and/or monocytes in response to *wt Bb*. Meninges from mice infected with *oppA2tn* did not display the same inflammatory phenotype observed in the *wt* infection, suggesting that by 4 wpi, the meningial microenvironment was not responding to *Bb* infection despite invasion of the lymphatic system at this time point.

Given immunoblot comparisons suggested significant changes in antibody response to our strains, we next sought to determine whether these differences reflected changes in immune cell populations in *wt-* and *oppA2tn-*infected mice. At 5 dpi and 4 wpi we performed complete blood count (CBC) analysis of circulating immune cells (**Figure 3B**; **Supplementary Figure 3A**). While comparisons between *wt* and *oppA2tn*-infected mice were not statistically significant, we observed trends of increased white blood cells, neutrophils, lymphocytes, monocytes, eosinophils, and basophils in *oppA2tn*-infected mice at 5 dpi. By 4 wpi, *wt*- and *oppA2tn*-infected mice had similar counts across all cell types. We further sought to evaluate site specific immune cell populations. As *oppA2tn* spirochetes were restricted to the skin or LN, we performed flow cytometry to characterize differences in immune cell populations in these tissues using flow cytometry. Characterization of immune cell populations during *Bb* infection in the skin were unsuccessful due to poor cell viability. To investigate LNs composition, we pooled a selection of ipsilateral LNs (PALN, AALN, SiLN) for flow cytometry. We chose timepoints to capture both early adaptive immune responses (5 dpi) and post-germinal center collapse (8 wpi) (28, 29) (**Figure 3C**). The only difference was a statistically insignificant decline in CD4^+^ T cell frequency at 5 dpi. While we may have seen slight masking effects by pooling LNs, overall, these data imply that either the flow cytometry approach is not sensitive enough to explain the differences in humoral responses or that these distinctions are not driven by changes in cell frequency.

### 3.4 Impaired hematogenous dissemination results in decreased *Bb-*specific IgG production

**Figure 4:**
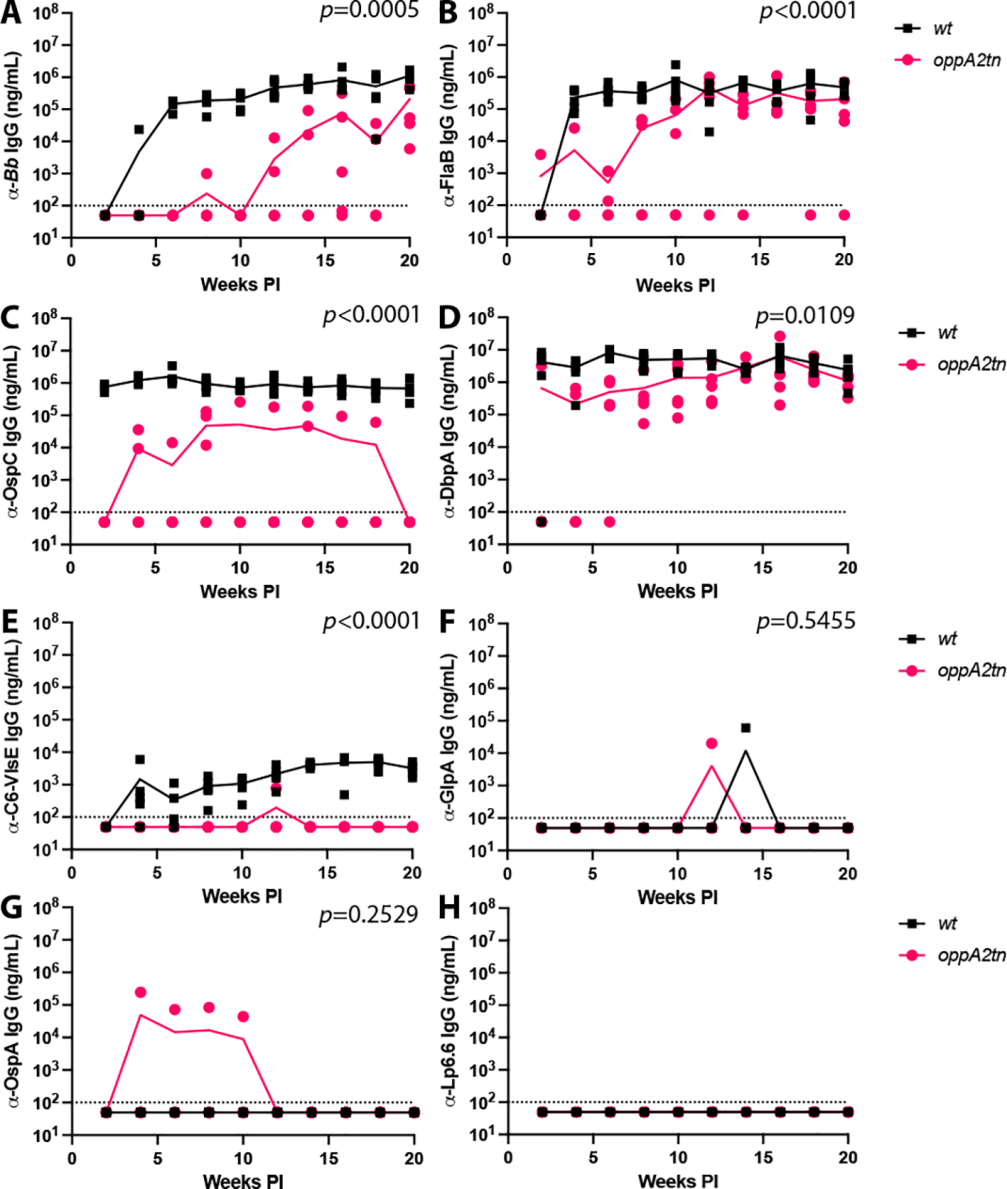
*oppA2* infected mice display a diminished *Bb*-specific IgG response. **(A-H)** IgG quantification by ELISA against **(A)** whole *Bb*, host-associated antigenic targets **(B)** FlaB, **(C)** OspC, and **(D)** DbpA, **(E)** C6 peptide of VlsE, as well as tick-associated antigenic targets **(F)** GlpA, **(G)** OspA and **(H)** lp6.6, which are down-regulated during infection. Symbols represent individual mice and the line represents the mean values at each timepoint. The dotted line represents the limit of detection (LOD) and samples that were undetectable by ELISA were plotted as LOD/2. Points represent individual mice, lines represent mean. *p* values were determined by 2-way ANOVA.

The observation that *oppA2tn*-infected mice showed diminished serological responses was intriguing. Therefore, we sought to quantify these differences via quantitative enzyme-linked immunosorbent assay (qELISA). We performed whole bacteria and antigen-specific qELISAs to measure IgG at 2 wpi to 20 wpi (**Figure 4**; **Supplementary Figure 4**; **Table 3**-Experiment 2). *Bb* lysates were used to measure overall *Bb*-specific antibody concentrations, while recombinant *Bb* proteins tracked antibody concentrations for key *Bb* antigens. FlaB is a constitutive, prominent antigen during *Bb* infection that is localized in the periplasmic space and thus is only available for immune sampling from dead, phagocytosed, or opsonized organisms (30–33). OspC, VlsE, and DbpA are RpoS-regulated, surface-localized lipoproteins expressed in the mammal during infection, with OspC downregulation paired with upregulation of VlsE occurring at approximately 2–3 wpi (34–36). GlpA, OspA, and Lp6.6 are tick phase proteins which are downregulated during initial colonization of the mammal (37–41).

Previous studies investigating anti-*Bb* antibody responses in mice have primarily shown quantitative total IgG antibody concentrations at 2–4 w timepoints ranging between 50-300 µg/ml (42–44) increasing to 400-700 µg/ml by 4w (45). Other studies have evaluated antibody toward specific *Bb* antigens, though non-quantitative methods were used or quantification was not directly reported, preventing direct comparison of our data for OspA, OspC, DbpA, and VlsE (7, 46–53). As expected, *wt-*infected mice demonstrated similar concentrations of IgG of all subclasses by 2–4 wpi for whole *Bb*, FlaB, OspC, and DbpA and VlsE antibodies (**Figures 4A–E**; **Supplementary Figures 4A–E**). Mice infected with *wt* also demonstrated a sustained *Bb*-specific IgG through 20w. There was no detectable antibody for the two proteins repressed by *Bb* in the host, OspA and lp6.6, for mice infected with the *wt* strain (**Figures 4F–H**; **Supplementary Figures 4F–H**). There was transient detection of antibody specific to the tick phase antigen GlpA in a single *wt*-infected mouse.

*OppA2tn*-infected mice had decreased, and often intermittent, *Bb*-specific IgG antibody in comparison to *wt*-infected mice (**Figures 4A–E**; **Supplementary Figures 4A–E**). Reactivity to whole *Bb* was negligible in three of the five *oppA2tn*-infected mice until 18-20 wpi and the remaining two mice were delayed until 10 wpi compared to *wt* at 2-4 wpi (**Supplementary Figure 4A**). This trend was maintained with anti-FlaB IgG (**Figure 4B**; **Supplementary Figure 4B**), although by 12 wpi FlaB antibody was near *wt*-infected levels in most mice. OspC antibody was both diminished and intermittent in *oppA2tn*-infected mice and waned to undetectable levels by 20 wpi, with only three of five mice generating detectable antibody throughout the time course (**Figure 4C**, **Supplementary Figure 4C**). We previously showed that the *oppA2tn* mutant strain expressed RpoS and RpoS-regulated proteins, including OspC, upon temperature shift and in host-adapted spirochetes, suggesting that OspC expression is not impaired in this strain (11). In contrast, anti-DbpA antibody in *oppA2tn*-infected mouse sera appeared to reach and sustain at *wt*-infected mouse sera levels in all mice despite an approximate 4–6 w delay compared to *wt*-infected mice (**Figure 4D**; **Supplementary Figure 4D**). VlsE specific antibodies against the invariant C6 peptide were only seen in a single mouse.

Two mice were found to have sporadic antibody responses against OspA and one mouse for GlpA within the first 12w, though no antibody against lp6.6 was detected (**Figures 4F–H**; **Supplementary Figures 4F–H**). It is important to note that antibodies against OspA result in bacterial clearance if these proteins are expressed in an immunocompetent host, resulting in strong selection pressure against expression of these antigens in the mammalian environment (54–56). It is unclear whether antibodies against lp6.6 specifically aid in *Bb* clearance, though the expression pattern of lp6.6 closely matches OspA (57). The mice that showed low levels of OspA antibody displayed culture positive tissues at 20 wpi (**Table 3** – Experiment 2), confirming that the intermittent antibody generated against OspA may have been stochastic. Furthermore, previous studies have shown low level OspA expression in some *Bb* infected tissues and OspA antibodies are detectable in human Lyme cases and in animal infection models despite OspA downregulation during mammalian infection (58–62). Importantly, Caimano et al. (13) showed that low levels of OspA antibody can be detected up to 20 wpi, though lack of quantification prevents a direct comparison with this data set. However, it was also shown that constitutive expression of OspA during infection results in a large and sustained increase in OspA antibody and bacterial clearance, which is not consistent with what we saw in our study (55). Taken together, these data show that *oppA2tn* infection induces delayed and inconsistent IgG response to *Bb* host-specific antigens (whole *Bb*, FlaB, OspC, DbpA). As there was not robust antibody titers against canonically down-regulated antigens, this suggests that *oppA2tn* mutant is not abnormally presenting RpoS-repressed antigens.

### 3.5 Spatial profiling does not expose variation in cell organization in skin or lymph nodes in response to defective hematogenous dissemination

Given that *oppA2tn* bacteria are restricted to skin and, subsequently, LNs for at least 8 wpi, we chose to perform spatial profiling on skin and LNs to gain a better understanding of potential perturbations to immune cell organization that could not be captured by flow cytometry or histopathologic characterizations. We used the MACSima Imaging Platform to visualize immune cell organization using staining protocols that allow for differentiation of macrophages, neutrophils, T cells, B cells, blood vessels, lymphatic vessels, epithelial, and mesenchymal tissue cell subsets, as well as Langerhans cells in the skin.

**Figure 5:**
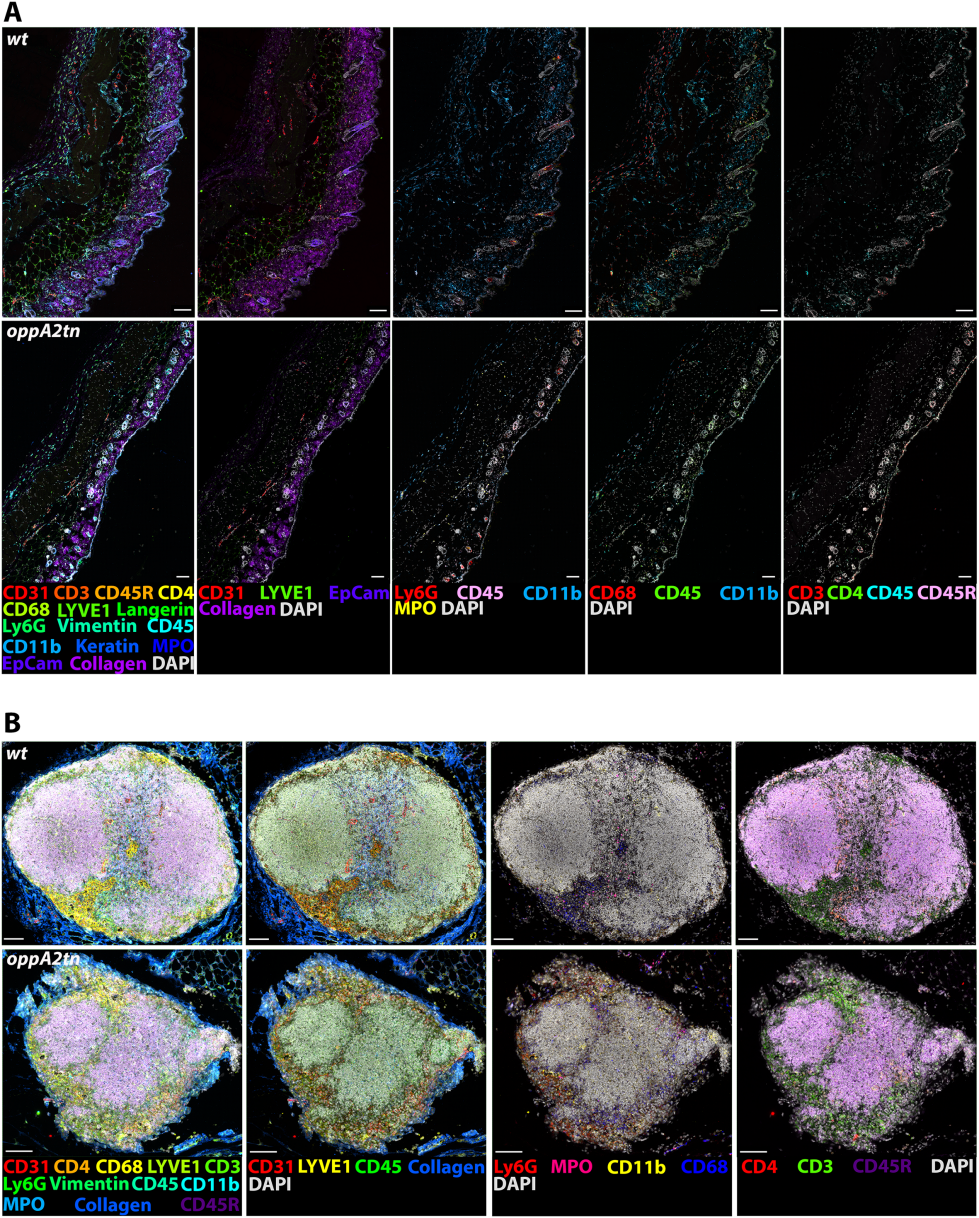
MACSima spatial profiling reveals subtle changes in cellularity in *Bb-*infected skin and LNs for *wt* and *oppA2tn*-infected mice. **(A)** Representative images of *wt*- (upper row) and *oppA2tn*-infected (lower row) mouse skin at 4 wpi. Note increased CD68 staining (in red, co-localized with CD11b in blue) indicative of macrophages in column 4. Scale bars represent 1,000 μm **(B)** Representative images of *wt*- (upper row) and *oppA2tn*-infected (lower row) mouse LN at 5 dpi. Note increased overall B cell density and increased CD45R (in lilac) staining indicative of B cells in *wt*-infected mice. CD3^+^ CD4^-^ cells were considered as CD8^+^ T cells. Scale bars represent 500μm.

In the skin, as expected we observed typical architecture and cellular organization with neutrophils, macrophages, and T cells organized around hair follicles in the epidermis (**Figure 5A**; **Supplementary Figure 5**). These cells act as first responders to perturbations in hair follicles (63). We were only able to detect Langerhans cells in a few skin samples; therefore, we were unable to determine any strain specific trends. At 5 dpi and 8 wpi, there were no obvious changes in cellular organization in tissue. However, at 4 wpi there appeared to be more macrophages present in *wt*-infected skin compared to *oppA2tn*-infected skin, suggesting increased macrophage infiltration and the potential for reduced clearance and/or sampling of *oppA2tn*.

In LN samples, we did not observe obvious changes in T or B cell zone organization (**Figure 5B**; **Supplementary Figure 6**). We stained CD4^+^ T cells to determine whether there were differences in the helper T cell subset. Attempts to use antibodies to detect either CD8α or CD8β were unsuccessful, therefore, we alternatively defined CD8^+^ T cells as CD45^+^ CD3^+^ CD4^-^ populations to infer presence of the cytotoxic T cell subset. We did not observe differences in either CD4^+^ or CD8^+^ T cells distributions between the *wt*- and *oppA2tn*-infected mice. However, there appeared to be increased B cell cellularity in *wt-*infected mice compared to *oppA2tn*-infected mice at 5 d, indicative of increased B cell expansion. At 4 wpi and 8 wpi, in similarly structured LN zones, there were some apparent differences in T and B cell cellularity between *wt*- and *oppA2tn*-infected animals, but was inconsistent between LN samples, likely due to “asynchronicity” in LNs similarly observed in histopathological assessments (**Figure 3A**). There were no striking differences between *wt*- and *oppA2tn*-infected mice LNs populations of classical dendritic cells, monocytes, neutrophils, or macrophages. Taken together, these data suggest that there may be increased B cell populations in *wt*-infected mice early in infection (5 dpi) compared to *oppA2tn-*infected mice, but this phenotype does persist throughout infection (4 wpi to 8 wpi).

### 3.6 Limiting *Bb* infection to intracutaneous dissemination results in dysregulation of host response

**Figure 6:**
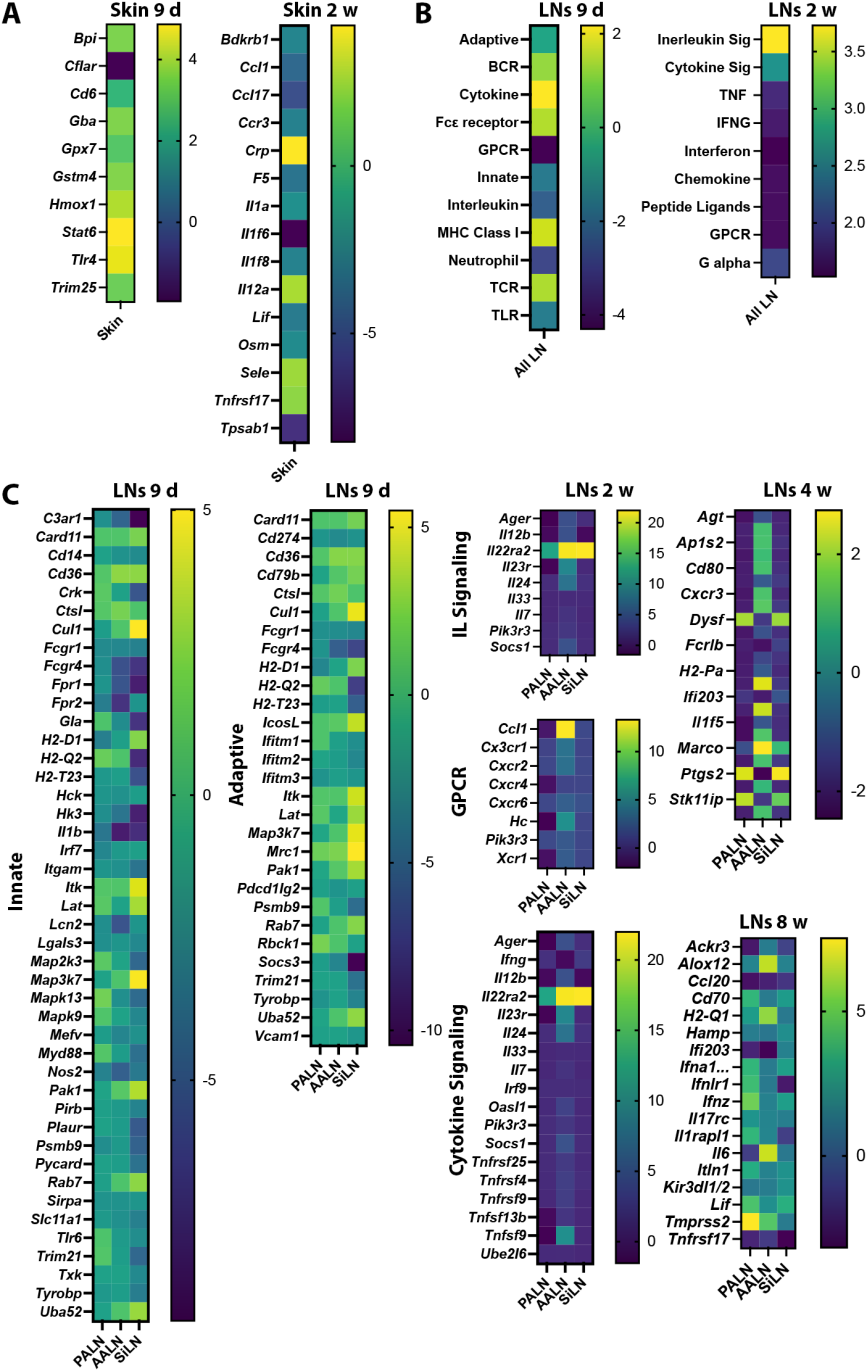
Transcriptional changes in skin and LNs for *wt* and *oppA2tn*-infected mice. **(A)** Heatmap showing fold changes for genes that were statistically significant (*p <*0.05) in skin at 9 dpi and 2 wpi. **(B)** Heatmap showing average fold changes across all LNs for pathways as determined by Reactome GO Pathway Analysis that were statistically significant (*p <*0.05) in LNs at 9 dpi and 2 wpi. **(C)** Heatmap of selected DE genes showing fold changes of *oppA2tn* versus *wt* for genes that were statistically significant (*p <*0.05) in LNs at 9 dpi and 2 wpi, gene sets are labeled according to their pathway in panel B.

Global and spatial analysis of immune cell populations provided little insight into the diminished antibody response to the *oppA2tn* mutant. Therefore, we sought to examine the functional state of immune cells during these infections by evaluating transcriptional differences in skin and LNs using the Nanostring nCounter murine host response panel. We collected tissue samples to represent early infection (9 dpi), germinal center formation (2 wpi), and at early- and late-dissemination stages (4 wpi and 8 wpi, respectively). Genes were considered differentially expressed (DE) if we observed a fold change (FC) > 2 and *p <*0.05 (**Supplementary Table 4**). In the skin, while PCA analysis suggested some sample clustering based on timepoint, these populations did not display high variability (**Supplementary Figure 7A**). Consistent with the PCA plot, we observed little DE when comparing *wt-*, *oppA2tn-*infected mice (**Figure 6A**; **Supplementary Table 4**). Only a handful of genes were DE in the skin at any time point. At 9 dpi, we observed DE in *Gpx7*, *Gstm4*, *Hmox1*, *Cd6*, *Trim25*, *Stat6*, *Bpi*, *Gba*, *Tlr4*, and *Cflar* (**Figure 6A**). All genes except *Cflar* were upregulated in the *oppA2tn* infection, suggesting a potential for increased antimicrobial response. Genes such as *Gpx7*, *Gstm4*, and *Hmox1* are involved in oxidative stress response (64–66). Other genes such as *Cd6*, *Trim25*, *Cflar*, and *Stat6* are involved in immune response regulation cascades (67–70). *Bpi* encodes the cationic antimicrobial peptide BPI (bactericidal/permeability-increasing protein), involved in antibacterial function against Gram negative bacteria (71). *Gba*, also known as glucocerebrosidase, is involved in glucosylceramide catabolism (72). While the majority of these have not been examined in *Bb* infection, TLR4 has been studied to some extent, showing that signaling through this canonical LPS-stimulated TLR is possible and its deletion impacts bacterial burdens and associated pathology (73, 74). However, the TLR4 ligand during *Bb* infection is unknown as *Bb* does not produce LPS. At 2 wpi, *IL1f6*, *Tpsab1*, *Ccl17*, *Ccl1*, *F5*, *Lif*, *Ccr3*, *Il1f8*, *Bckrb1*, *Osm*, *Il1a* expression was significantly lower in *oppA2tn*-infected mouse skin compared to *wt*-infected mice whereas four genes (*Tnfrsf17*, *Sele*, *Il12a*, *Crp*) were upregulated (**Figure 6A**). Several of these downregulated genes (*Il1f6*, *Il1f8*, *Il1a*, *Il12a*, *Osm*, *Lif*, *Ccl17*, *Ccl1*, *Ccr3*) are implicated in proinflammatory signaling and chemotaxis (75–79) and may result in reduced signaling and cellular infiltration to the skin during *oppA2tn* infection. At 4w and 8w, we did not observe any significant changes in gene expression between *wt* and *oppA2tn* infections.

Contrary to the skin, we saw robust DE in the LNs, specifically at 9 dpi and 2 wpi (**Figures 6B, C**; **Supplementary Table 4**). We evaluated LNs individually, in the event that pooling LNs samples resulted in a lower resolution analysis. When we visualized all LN samples on a PCA plot (**Supplementary Figure 7B**), we did not see distinct clustering of sample sets. However, when we visualize a single LN set at a single time point (**Supplementary Figure 7C**), samples showed discrete clusters. We observed the greatest impact on gene expression in *oppA2tn*-infected animals compared to *wt*-infected animals at 9 dpi, followed by 2 wpi; later timepoints (4 wpi and 8 wpi) showed few overall changes. These trends are consistent with reports that germinal center responses to *Bb* wane after 2w of infection (28, 29).

At 9 dpi, we observed downregulation of innate and adaptive immune responses in *oppA2tn*-infected mice (as determined by Reactome GO Pathway Analysis) (**Figures 6B, C**). Our pathway analysis also showed downregulation in specific pathways such as interleukin signaling, TLR cascade, and GPCR downstream signaling (**Figure 6B**). Of these genes, we observed a collection of genes that previous *Bb* infection studies have investigated such as *MyD88*, *Cd14*, *Itgam*, *Irf7*, *Il1b*, *Nos2*, and *Tlr6* (**Figure 6C**). MyD88 is a critical adaptor protein involved in signal transduction via TLR and IL-1R binding (80). In the context of *Bb* infection, MyD88 is involved in phagocytosis and upregulation of proinflammatory responses in macrophages (81, 82), T cell differentiation in lymphoid tissues (83), and *Bb* clearance *in vivo* (84). Similarly, a role for CD14 in *Bb* phagocytosis and cell signaling has also been shown (85–89). Furthermore, *Itgam*, encoding CD11b (which forms a heterodimer with CD18 to become complement receptor 3; CR3) is implicated in *Bb* phagocytosis (85, 88, 90, 91). Once *Bb* is phagocytosed, *Bb* nucleic acids can be released during digestion, activating endosomal TLRs (such as TLR7 and TLR8) and leading to transcription of Irf7 (92–95). Irf7 expression and subsequent signaling cascade leads to type I interferon production and antimicrobial response (96). While less studied in the context of *Bb* infection, IL-1β has previously been shown to be upregulated during *Bb* inoculation in neutrophils, macrophages, and splenocytes (97). Furthermore, *Nos2*, encoding inducible nitric oxide synthase (iNOS) has been shown to be upregulated in macrophages in *Bb* infected joints and hearts (98). TLR6, however, has little impact on *Bb* host response but has been shown to heterodimerize with TLR2 which recognizes *Bb* lipoproteins (99, 100).

At 2 wpi, we observed similar gene expression patterns between *wt* and *oppA2tn* infections in PALN and SiLN; however, the AALN of *oppA2tn*-infected animals showed greater overall differences, notably upregulation of expression in genes relating to GPCR signaling and cytokine signaling (**Figures 6B, C**). DE genes such as *Ifng*, *Ccl3*, *Cxcr2*, *Cxcr4*, *Cxcr6*, and *Cx3cr1* have previously been examined in the context of *Bb* infection. Many studies have investigated the role of *Ifng* (encoding Interferon-γ; IFN-γ) during *Bb* detection, and host pathologies in Lyme arthritis and carditis (101–107). Ccl3, otherwise known as MIP-1α, has also been implicated in *Bb* response and associated pathologies (108–112). The chemokine receptors Cxcr2, Cxcr4 and Cxcr6 have been minimally investigated in *Bb* infection, but their upregulation has been shown to have roles in Lyme arthritis and carditis (113), response to *Bb* infection (113, 114), and protection against joint *Bb* infiltration (115). Similarly, the “M2” macrophage marker Cx3cr1 has also been understudied, but previous work has shown that its expression is temporally regulated during *Bb* infection in microglia where it decreases within 24h post-infection (116). This study further suggested that *Bb* infection increases pro-inflammatory, “M1” macrophage signatures early in microglia infection. This regulatory profile suggests that *oppA2tn* infection at 2 wpi may either “catch up” to *wt* infection transcriptional responses or the *oppA2tn* infection may have some residual inflammation after resolution of inflammation observed at 9d.

At 4 wpi, we were only able to identify four genes (*Agt*, *Cxcr1*, *Cxcr3*, *Cxcr6*) that were statistically significant in *oppA2tn*-infected mice compared to mice infected with *wt* (**Supplementary Table 4**). These genes are either involved in chemokine signaling or macrophage, neutrophil, and T cell infiltration (117–120). We observed downregulation of these four genes in the PALN and SiLN of *oppA2tn*-infected mice, however expression of *Cxcr3* and *Cxcr6* were slightly elevated in AALN in the same mice (**Supplementary Table 4**). By 8w, we only detected six DE transcripts between *wt* and *oppA2tn*-infected mice, all involved in cytokine signaling (*Cd70*, *Ifnlr1*, *Il1rapl1*, *Il6*, *Lif*) (**Supplementary Table 4**). Of these genes, *Il6* is the only studied in the context of *Bb* infection, where it has primarily been implicated in Lyme disease pathologies (101, 121–123). Together, these data suggest that the greatest differences during infection with the *oppA2tn* mutant are apparent during early infection, the effects of which likely contribute to a lack of serological response during prolonged *Bb* infection.

## 4 Discussion

While some aspects of *Bb* systemic dissemination within the host are well defined, many questions remain concerning the nuances of this complex process. Herein, we sought to characterize the *oppA2tn* mutant 1) to define this intracutaneous dissemination model longitudinally and 2) to understand how this model impacts immune detection and response against the spirochete. For *Bb*, the transition from the feeding vector to the naïve host requires complex transition of gene expression programming; these changes are primarily driven by the transition from c-di-GMP-driven gene expression (40, 124, 125) to initiation of the Rrp2/RpoN/RpoS sigma factor cascade program (126). This host adaptation process facilitates bacterial expansion and migration out of the midgut of the tick into the host, as well as host dissemination and immune evasion once infection is established in the host (5, 126, 127). To date, we have not found aberrant protein expression in the *oppA2tn* mutant either *in vitro* or *in vivo* that could contribute to the phenotypic features seen in this study or in our previous study (e.g., OspA, OspC) (11) These data suggest that this mutant’s phenotype is not due to dysregulation of the global host-adaptation processes, though it is unclear how loss of a peptide binding protein results in abrogation of hematogenous dissemination. We previously hypothesized OppA2 could interface with *Bb*’s chemotaxis array, acting as a general peptide sensor to the external milieu in addition to acquiring peptides for replenishing amino acid pools (11), and abrogation of *Bb*’s chemo-sensory system would impact the spirochete’s ability to disseminate. Indeed, a recent study examining the importance of the chemosensory-like histidine kinase CheA_1_ observed similar dissemination and skin viability phenotypes that we observed in *oppA2tn* infections, however this mutant also displayed tick related phenotypes and skin colonization outside of the inoculation site was not evaluated (128). Given that *oppA2tn* spirochetes appear to proliferate and survive within the mammal up to 20 wpi, we infer that the other four OppAs expressed in the mammalian environment display enough functional overlap to obtain amino acids.

Our infection studies confirmed that, given enough time, *oppA2tn* spirochetes can migrate throughout the skin, into lymph nodes, and eventually distal organ sites, though this process takes significantly longer than hematogenous dissemination (20w vs 2w, respectively) and is not as pervasive with respect to distal organs. Normally, spirochetes are detected in the blood approximately 3–7 dpi, which also parallels with their detection in CSF (27). Interestingly, this hematogenous dissemination stage is only transient, and, in contrast with its relapsing fever counterpart, *Bb* does not replicate to high densities during this process (129, 130). During spirochetemia, spirochetes will tether to endothelial cells via host ligands such as fibronectin and glycosaminoglycans and extravasate into tissue establishing localized foci of infection throughout organs (129). Migration outward from these foci results in a fully disseminated infection by approximately 2 wpi. While we have had evidence that *Bb* likely utilized the lymphatic system as a dissemination route due to their colonization of LNs (26, 28), hematogenous dissemination had previously cloaked any meaningful assessment of systemic dissemination dynamics via this route. On the surface, this delayed dissemination process appears to dampen the overall host response, specifically bacteria-specific serological responses, suggesting that it is dissemination to distal site(s) that drives the host response. However, parallel analyses will need to be conducted with additional mutants which display dissemination phenotypes to conclusively evaluate this dynamic. We were also able to demonstrate that both the innate and adaptive immune responses broadly influence both intracutaneous migration and the ability to reach distal organs. At this time, it is unclear whether the ability for the host immune response to dampen cutaneous dissemination is due to a defect in the *oppA2tn* mutant, or if this control is common to *wt* infections as well. During the course of a *wt* infection, the ability for the bacteria to hematogenously disseminate with such impunity would veil the ability for the immune system to curtail cutaneous migration, though the ability of the immune system to limit bacterial burdens during *wt* infection (131, 132) suggests these pressures are felt no matter the route of dissemination.

Previous studies have shown that mice inoculated with heat-killed *Bb* generate very little serological response (42) despite activating innate immune responses (106, 133, 134). Furthermore, mutants with significant defects (*e.g.* motility or chemotactic defects) are quickly cleared by the innate immune system and elicit weak or no serological responses (135–137). We had previously shown that *oppA2tn* burdens in the skin were commensurate with *wt* burdens (11), however it is possible that there are lower burdens early during infection, and, given the limited distal tissue colonization, there is sure to be a lower overall systemic burden that may contribute to reduced serological responses. Attempts to investigate this phenomenon unveiled little difference in pathology or in immune cell composition or their spatial organization. These data suggest the difference in immune response to *oppA2tn* may reflect a difference in functional response, not a population variance. Indeed, transcriptomic analyses uncovered changes in genes both relating to innate and adaptive immune processes potentially involved.

Our transcriptional analysis of skin and LNs provided some insight into dysregulated host responses responsible for differences in humoral response. In the skin, we primarily observed differences at earlier infection timepoints, implying the skin reaches a level of “homeostasis” in later infection time points (4 wpi and 8 wpi) despite persistent *Bb* infection. At 9 dpi, some signaling and antimicrobial response related genes were upregulated in the *oppA2tn*-infected mice compared to *wt*-infected mice in the skin. This gene regulation trend in similarly related genes reversed to downregulation at 2w, implying that at 9 dpi there is increased host response to *oppA2tn* in the skin that decreases by 2w. While we have not observed significant changes in bacterial burden in the skin with the *oppA2tn* infection previously (11), there may be instances of increased innate anti-*Bb* response that is more quickly resolved compared to *wt* infection.

In LNs, we observed an overall decrease in transcripts related to innate and adaptive immunity at 9 dpi in *oppA2tn*-infected mice, despite increased signaling transcripts in the skin. These contrasting immune responses may represent a possible disconnection between antigen recognition in *oppA2tn-*infected tissue and antigen presentation and response in lymph node sites. Notably, genes involved with *Bb* recognition and response to antigen recognition (*MyD88*, *Cd14*, *Itgam*, *Irf7*, *Il1b*, *Nos2*, *Tlr6*) were downregulated at this timepoint, clearly indicating differences in LN cell activation in response to *oppA2tn*-infected mice compared to *wt*-infected mice. At 2 wpi, there were decreases in pro-inflammatory molecules such as IFN-γ and Ccl3 in response to the *oppA2tn* infection compared to *wt*-infected mice showing that response to infection at 2 wpi is still impacted. The detachment of antigen recognition in *oppA2tn*-infected sites and presentation in lymphoid tissue would establish a deficit in innate and adaptive immune response, possibly explaining the deficiencies observed in antibody production we observed here. There were fewer differences in DE genes at 4 wpi and 8 wpi in *oppA2tn*-infected mice, suggesting that early recognition of *Bb* in tissue and proper response to *Bb*-associated PAMPs in lymphoid tissue may be critical for establishing strong humoral responses. It is likely that spatial transcriptomics would provide greater resolution in understanding which cell populations and pathways are influencing this abrogated response.

Despite having a more complete understanding of this non-hematogenous dissemination model, it is still unclear how *oppA2tn* infection results in such an altered serological response given that antigen is theoretically present and abundant over the entire course of infection. To our knowledge, the *oppA2tn* mutant is the only mutant to date that survives within the host in the absence of hematogenous dissemination, and we hope to use this infection model to gain greater insights into intracutaneous and lymphatic migration without the confounding influence of hematogenous dissemination. Interestingly, our data suggest that colonization of distal organ sites may drive antibody response during infection, and containment of the bacteria within the skin allows the bacteria to subvert those detection mechanisms. It is possible that in a site like the joints, a favored niche for *Bb*, bacterial debris can accumulate at levels that provide robust antigen presentation and B cell response. As evidence exists for residual, persistent peptidoglycan in the joint (138, 139), it is likely other *Bb*-associated PAMPs may also be found. Furthermore, we did not recover viable *oppA2tn* from the heart, even at 20 wpi, which could suggest another distal site may be critical for antigen sampling.

Why then would *Bb* choose hematogenous dissemination at the expense of a robust serological response? Indeed, deposition and acquisition of spirochetes during the cycle occur at the skin interface, making hematogenous dissemination and colonization of internal organs seem non-essential to these processes. Likely, the advantage gained is time. If the goal after transmission from the tick to mammal is to then be acquired again, complete colonization of the skin is imperative to improve these chances. While non-hematogenous dissemination eventually expands to all skin sites, this takes approximately 8w, while hematogenous dissemination accomplishes this task in a third of the time. Seasonal activity of the *Ixodes* tick has shown that nymphal questing can precede larval questing by as little as a month (140). *Ixodes* larvae require a bloodmeal on an infected mouse to become infected, so within a feeding season infected nymphs must deliver *Bb* to new cohorts of naïve mammals. This provides an infected bloodmeal for the subsequent cohort of larvae to acquire *Bb* and generate a new cohort of infected nymphs. Notably, *Peromyscus leucopus* (the primary reservoir species for *Bb*) life expectancy is estimated to be less than 1 year due to predation, food limitation, and weather conditions (141). These data suggest that few infected animals survive to the next annual feeding cycle and that it is critical for *Bb* transmission and acquisition to be completed within the active feeding windows of the larvae and nymphs each year (141). The timing of this cycle may make it advantageous for *Bb* to sacrifice immune detection for speed in its endeavor to propagate within the enzootic cycle.

## Data Availability

The original contributions presented in the study are included in the article/**Supplementary Material**. The data discussed in this publication have been deposited in NCBI's Gene Expression Omnibus and are accessible through GEO Series accession number GSE330239. Further inquiries can be directed to the corresponding author.
