# Supplementary material for: Characterizing the impact of intracutaneous dissemination on host responses during Borrelia burgdorferi infection
Source: Front Immunol. 2026 Jun 5;17:1850665. doi: 10.3389/fimmu.2026.1850665 (PMC13278888; doi:10.3389/fimmu.2026.1850665)
Supplement: Supplementary file 2 [file DataSheet1.pdf]

Supplementary Material

Characterizing the impact of intracutaneous dissemination on host responses during *Borrelia burgdorferi* infection

Samantha Crane[1], Amira-Nuriya McKinney[1], Clayton Jarrett[1], Chad S. Clancy[2], Olof Rickard Nilsson[1,#], Kelly L. Hawley[3-5], Ashley Groshong[1*]

[1]Laboratory of Bacteriology, Rocky Mountain Laboratories, Division of Intramural Research, National Institute of Allergy and Infectious Diseases, National Institutes of Health, Hamilton, Montana, USA

[2]Rocky Mountain Veterinary Branch, Rocky Mountain Laboratories, Division of Intramural Research, National Institute of Allergy and Infectious Diseases, National Institutes of Health, Hamilton, Montana, USA

[3]Departments of Pediatrics, Medicine, and Immunology, University of Connecticut School of Medicine, Farmington, CT, USA

[4]Department of Research, Connecticut Children's Research Institute, Hartford, CT, USA

[5]Divisions of Research, and Infectious Diseases and Immunology, Connecticut Children's, Hartford, CT, USA

***Correspondence:** Ashley Groshong, Laboratory of Bacteriology, Rocky Mountain Laboratories, Division of Intramural Research, National Institute of Allergy and Infectious Diseases, National Institutes of Health, Hamilton, Montana, USA. 1.406.802.6421. ashley.groshong@nih.gov

# Current affiliation: Department of Microbiology, Weber State University, Ogden, UT, USA

Contents:

Supplementary Tables 1-3

Supplementary Figures 1-6

Supplementary References

Supplementary Table 1. Strains and plasmids used in this study

Strain/Plasmid	Description	Antibiotic Resistance	Reference
B. burgdorferi			
BbG100	Wild-type strain B31 5A18 NP1 *bbe02::AphI* (*wt*)	Kan	(1)
BbG117	B31 5A18 NP1 *bb0329* transposon mutant (*bb0208tn*)	Kan/Gent	(2)
E. coli			
Stellar	*F–, endA1, supE44, thi-1, recA1, relA1, gyrA96, phoA, Φ80d lacZΔ M15, Δ (lacZYA - argF) U169, Δ (mrr - hsdRMS - mcrBC), ΔmcrA, λ–*	N/A	Clonetech
BL21 (DE3)	*F^- ompT $hsdS_B$ ($r_B^- m_B^-$) gal dcm (DE3)*	N/A	Invitrogen
C41 (DE3)	*F- ompT hsdSB (rB-mB-) gal dcm (DE3)*	N/A	Lucigen
Plasmids			
pET28a	Expression construct; N-terminal 6xhis tag	Kan	Novagen
pG282A	pET28a::FlaB (aa 1-337)	Kan	This study
pG283A	pET28a::OspC (aa 19-211)	Kan	This study
pG292A	pET28a::OspA (aa 17-274)	Kan	This study
pG293A	pET28a::lp6.6 (aa 18-69)	Kan	This study
pG294A	pET28a::GlpA (aa 1-521)	Kan	This study
pJSB782	pProEX HTa::DbpA (aa 26-187)	Amp	(3)

Supplementary Table 2. Oligonucleotide primers used in this study

Designation	Sequence (5'-3')	Purpose	Reference
5' flaBopt28a	**GCGCGGCAGC*CATATG***ATGATTATTAACCACAACACTAGCGCGATTA	Cloning	This study
3' flaBopt28a	**GGTGGTGGTG*CTCGAG***TTAGCGCAACAGTGAAAGGACGTATTG	Cloning	This study
5' -SSospCopt28a	**GCGCGGCAGC*CATATG***AATAACTCGGGGAAAGACGGAAACACAA	Cloning	This study
3' ospCopt28a	**GGTGGTGGTG*CTCGAG***TTAAGGCTTCTTCGGGGATTCCG	Cloning	This study
5' glpAopt28a	**GCGCGGCAGC*CATATG***AACAATAATAAAGAGACCAAACTTAAGGACC	Cloning	This study
3' glpAopt28a	**GGTGGTGGTG*CTCGAG***TTAAATAAGGTATTTCTTCGAAATCTCAAGGAAGTTCTTGA	Cloning	This study
5' -SSlp6.6opt28a	**GCGCGGCAGC*CATATG***GAGACCACGCGCATTTCAGACG	Cloning	This study
3' lp6.6opt28a	**GGTGGTGGTG*CTCGAG***TTACTTTTTCATACTTTTGGTCATAGGCTGTTTCA	Cloning	This study
5' -SSospAopt28a	**GCGCGGCAGC*CATATG***AAACAAAATGTAAGCAGTCTTGATGAGAAAAATTCC	Cloning	This study
3' ospAopt28a	**GGTGGTGGTG*CTCGAG***TTACTTAAGTGCGTTTTTAATCTCGTCTAATTTGG	Cloning	This study

Bold denotes overlap sequence for InFusion cloning. Italics denotes restriction sites.

Supplementary Table 3. Percent skin samples infected by location

	**4 w**		**8 w**			**20 w**		**MyD88**		**SCID**	
Skin site location*	*wt*	*oppA2tn*	*wt*	*oppA2tn*	Abbrev. site location**	*wt*	*oppA2tn*	*wt*	*oppA2tn*	*wt*	*oppA2tn*
E	100	0	100	80	E	100	89	100	60	100	90
IS	100	100	100	100	IS	100	100	100	100	100	100
1	100	100	100	100	1	40	67	80	100	100	70
2	80	0	100	100	ND						
3	80	0	100	100	2	70	56	90	100	100	70
4	60	40	100	100	ND						
5	80	100	100	100	ND						
6	100	20	100	100	ND						
7	100	80	80	100	ND						
8	100	20	100	100	ND						
9	100	100	100	100	ND						
10	100	100	100	100	ND						
11	100	80	80	80	ND						
12	80	80	100	60	3	80	89	100	100	100	70
13	100	80	80	100	ND						
14	100	80	100	100	4	100	89	100	100	100	60
15	80	0	60	40	5	100	89	100	70	100	80
16	100	0	100	80	ND						
17	100	0	100	80	6	100	44	100	70	100	50
18	80	0	80	80	ND						
19	80	20	60	80	ND						
20	100	40	100	100	ND						
21	80	0	100	80	ND						
22	80	0	100	100	ND						
23	100	40	100	100	ND						
24	100	20	100	100	ND						
25	100	0	100	80	ND						
26	100	0	100	100	ND						
27	80	60	80	100	7	70	89	100	60	100	50
28	100	20	100	80	ND						
29	100	0	100	80	8	100	44	100	70	100	70

*See Supplemental Figure 1A for sampling map

**See Supplemental Figure 1B for sampling map, sites have been placed at the correlating row for the full sampling data set, ND – not done

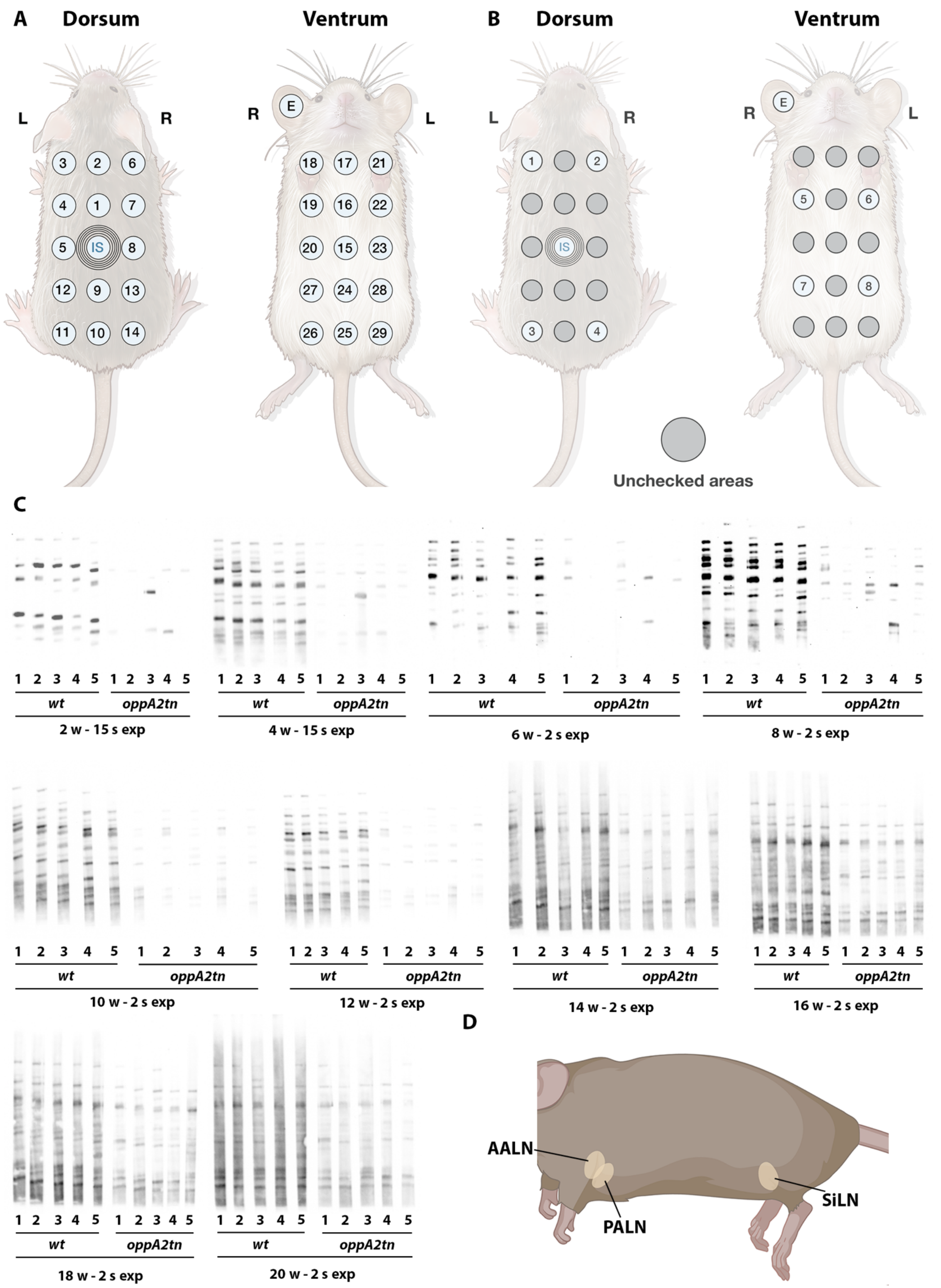
A
Dorsum
Ventrum
L
R
B
Unchecked areas
IS
E
C
wt
oppA2tn
2 w - 15 s exp
4 w - 15 s exp
6 w - 2 s exp
8 w - 2 s exp
10 w - 2 s exp
12 w - 2 s exp
14 w - 2 s exp
16 w - 2 s exp
18 w - 2 s exp
20 w - 2 s exp
D
AALN
PALN
SiLN

Supplementary Figure 1. Culture sites and individual immunoblots for longitudinal infection studies. **(A-B)** Skin sampling maps for **(A)** 4 w and 8 w culture data and **(B)** 20 w and immunocompromised infections. **(C)** Sera immunoblots of mice quantified in ELISA studies from 2 w to 20 wpi. **(D)** Location of PALN, AALN, and SiLN collected in this study.

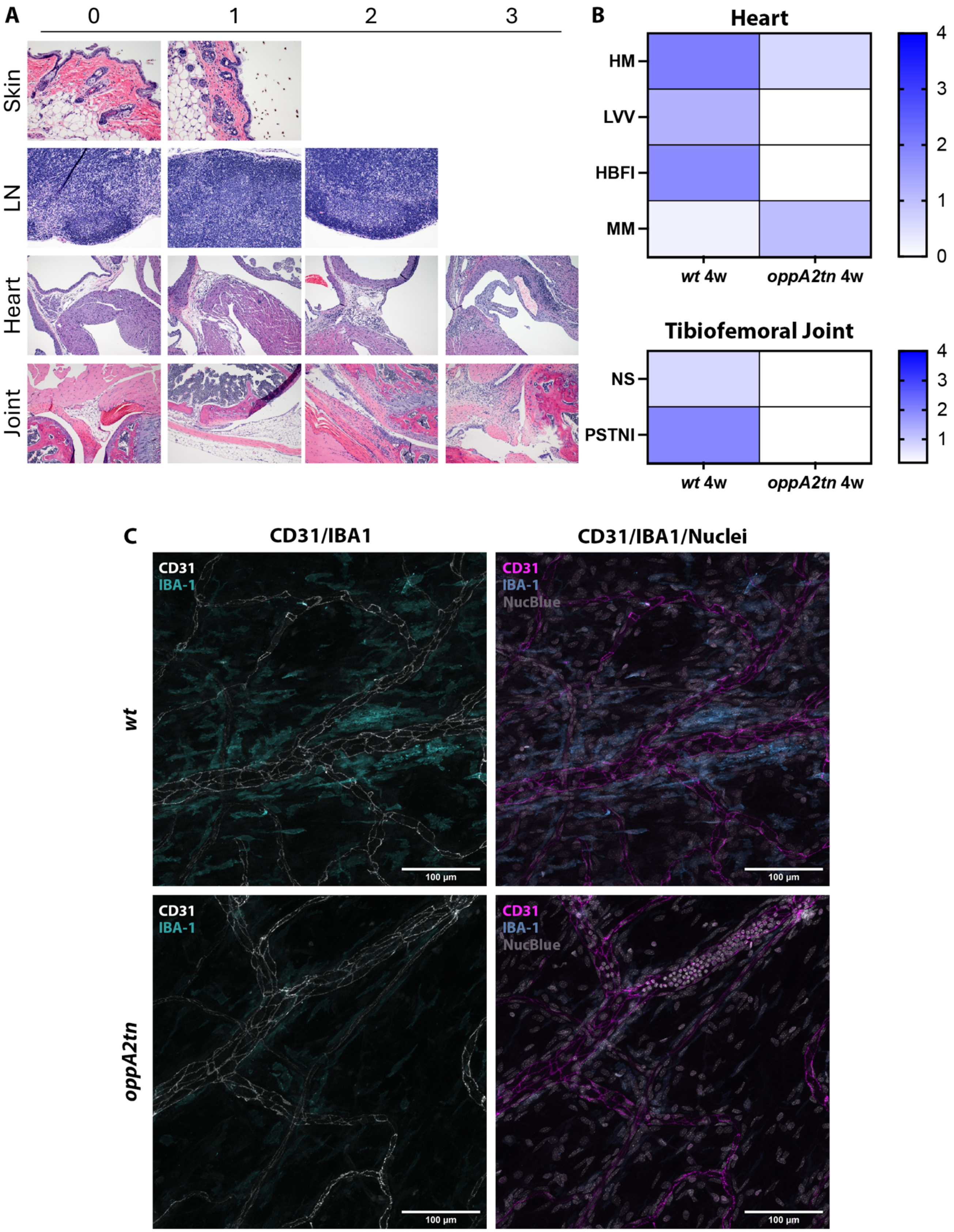
A
0
1
2
3
Skin
LN
Heart
Joint
B
Heart
HM
LVV
HBFI
MM
wt 4w
oppA2tn 4w
4
3
2
1
0
Tibiofemoral Joint
NS
PSTNI
wt 4w
oppA2tn 4w
4
3
2
1
C
CD31/IBA1
CD31/IBA1/Nuclei
wt
oppA2tn
CD31
IBA-1
NucBlue
100 µm

Supplementary Figure 2. (A) Scoring matrix for histopathological analysis with representative H&E images. **(B)** Heatmap of histopathological scores for heart and tibiofemoral joint, scores are averaged among cohorts. HM = histiocytic myocarditis, LVV = large vessel vasculitis, HBFI = heart base fibro-fatty inflammation, MM = myocardial mineralization, NS = neutrophilic synovitis, PSTNI = peri-articular soft tissue neutrophilic inflammation. **(C)** Representative images of stained meninges (CD31, IBA-1, NucBlue) at 4 wpi.

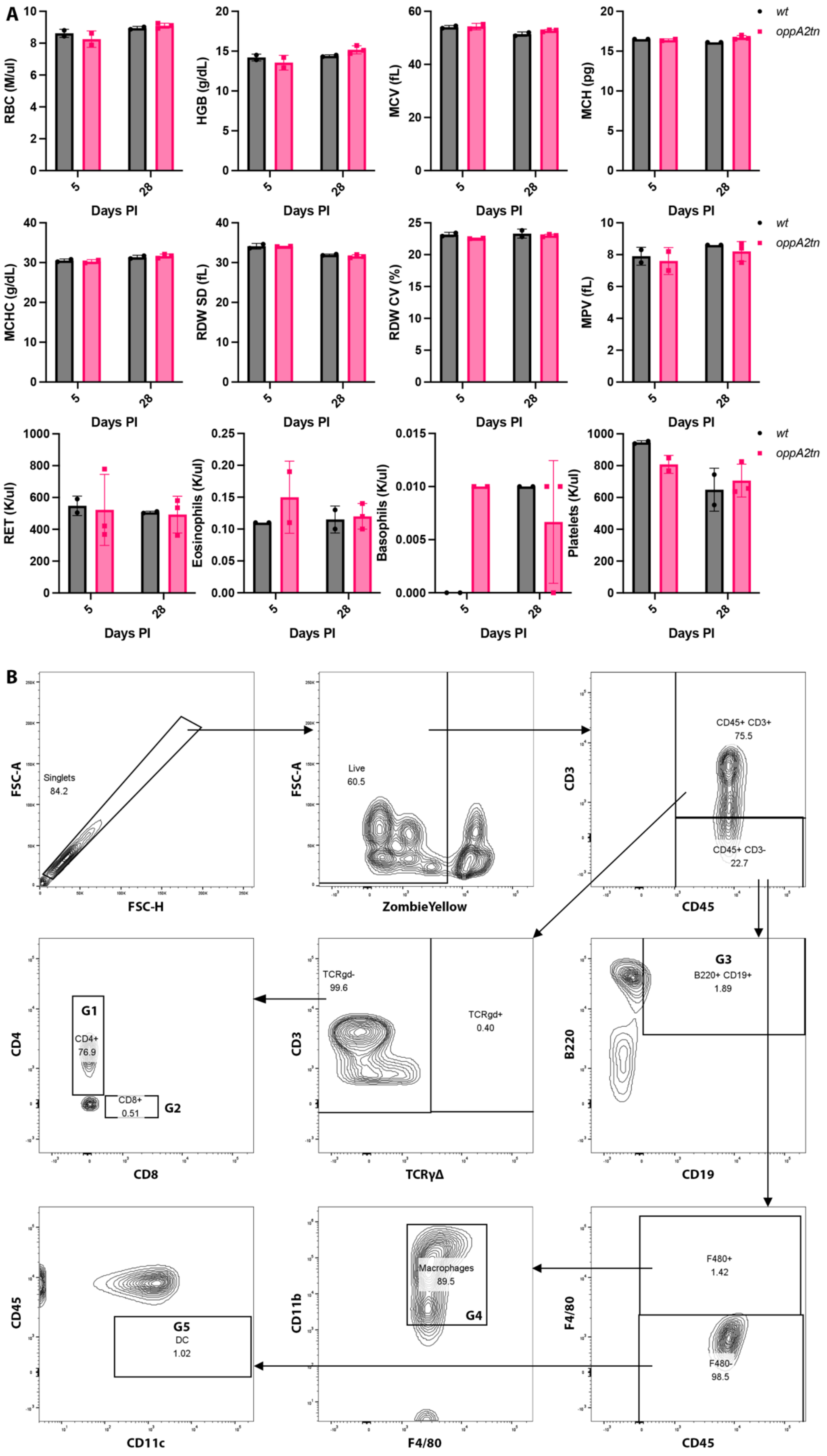
A
RBC (M/ul)
HGB (g/dL)
MCV (fL)
MCH (pg)
MCHC (g/dL)
RDW SD (fL)
RDW CV (%)
MPV (fL)
RET (K/ul)
Eosinophils (K/ul)
Basophils (K/ul)
Platelets (K/ul)
Days PI
wt
oppA2tn
B
FSC-A
FSC-H
Singlets
84.2
ZombieYellow
Live
60.5
CD3
CD45
CD45+ CD3+
75.5
CD45+ CD3-
22.7
CD4
CD8
G1
CD4+
76.9
G2
CD8+
0.51
TCRγΔ
TCRgd-
99.6
TCRgd+
0.40
B220
CD19
G3
B220+ CD19+
1.89
CD11c
G5
DC
1.02
CD11b
F4/80
Macrophages
89.5
G4
F4/80
F480+
1.42
F480-
98.5

Supplementary Figure 3. (A) CBC results for red blood cells (RBC), hemoglobin (HGB), mean corpuscular volume (MCV), mean corpuscular hemoglobin (MCH), MCH concentration (MCHC), red cell distribution width – standard deviation (RDW SD), RDW– coefficient of variation (RDW CV), mean platelet volume (MPV), reticulocytes (RET), eosinophils, basophils, and platelets as 5 d and 4 w (28 d). Points represent individual mice, bars represent mean, and error bars represent SEM; no statistical significance was found for panels B-C using 2-way ANOVA. No statistical significance was found using 2-way ANOVA. **(B)** Gating strategy used for flow cytometry experiments. Gates G1-G5 are final gates used for $CD4^+$ T cells, $CD8^+$ T cells, B cells, macrophages, and dendritic cells respectively. Gating strategy was determined using fluorescence minus one (FMO) samples.

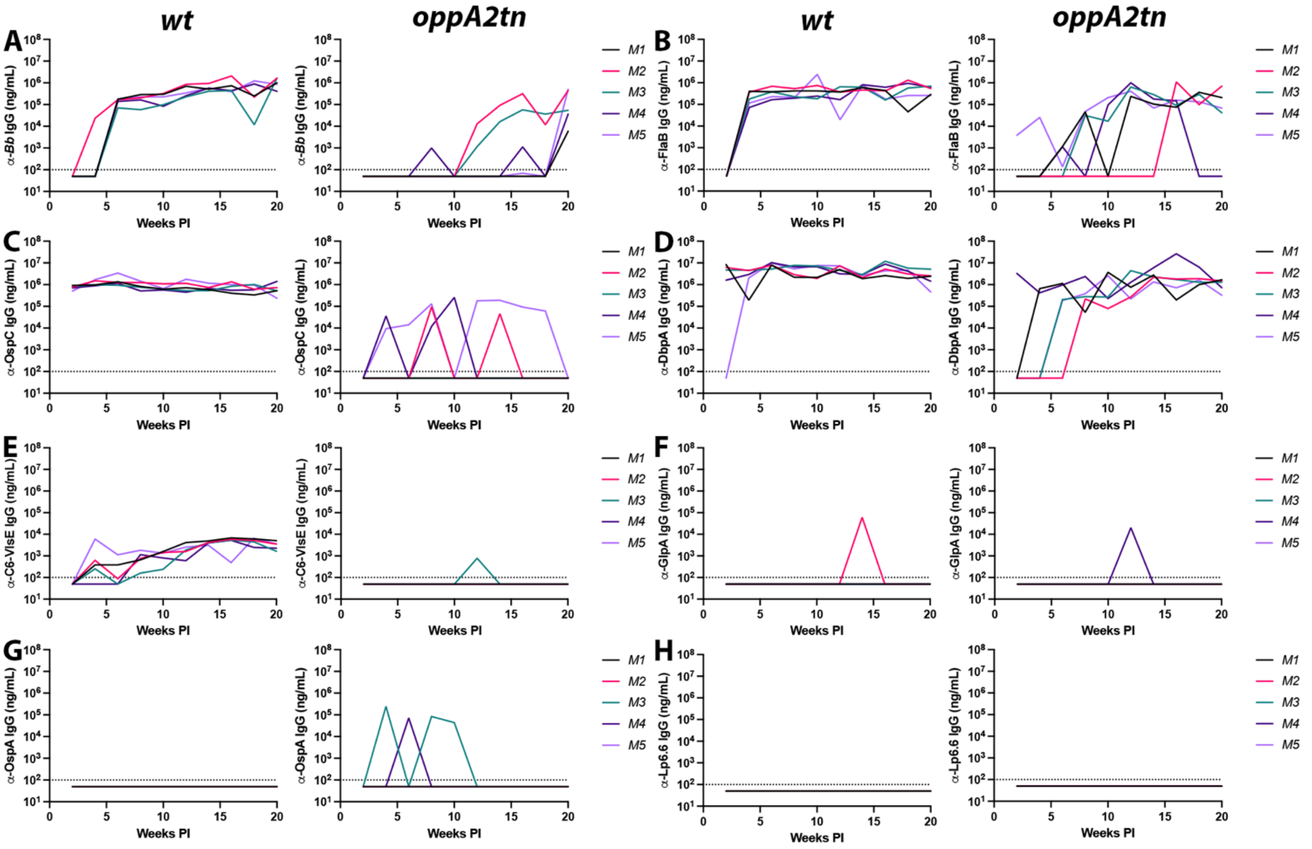

Supplementary Figure 4. Individual *oppA2tn* infected mice display intermittent, diminished serological responses against key antigens. (A-B) IgG quantification by ELISA against **(A)** whole *Bb*, host-associated antigenic targets **(B)** FlaB, **(C)** OspC, and **(D)** DbpA, **(E)** C6 peptide from VlsE, as well as tick-associated antigenic targets **(F)** GlpA, **(G)** OspA and **(H)** lp6.6, which are down-regulated during infection. Lines represent individual mice. The dotted line represents the limit of detection (LOD) and samples that were undetectable by ELISA were plotted as LOD/2.

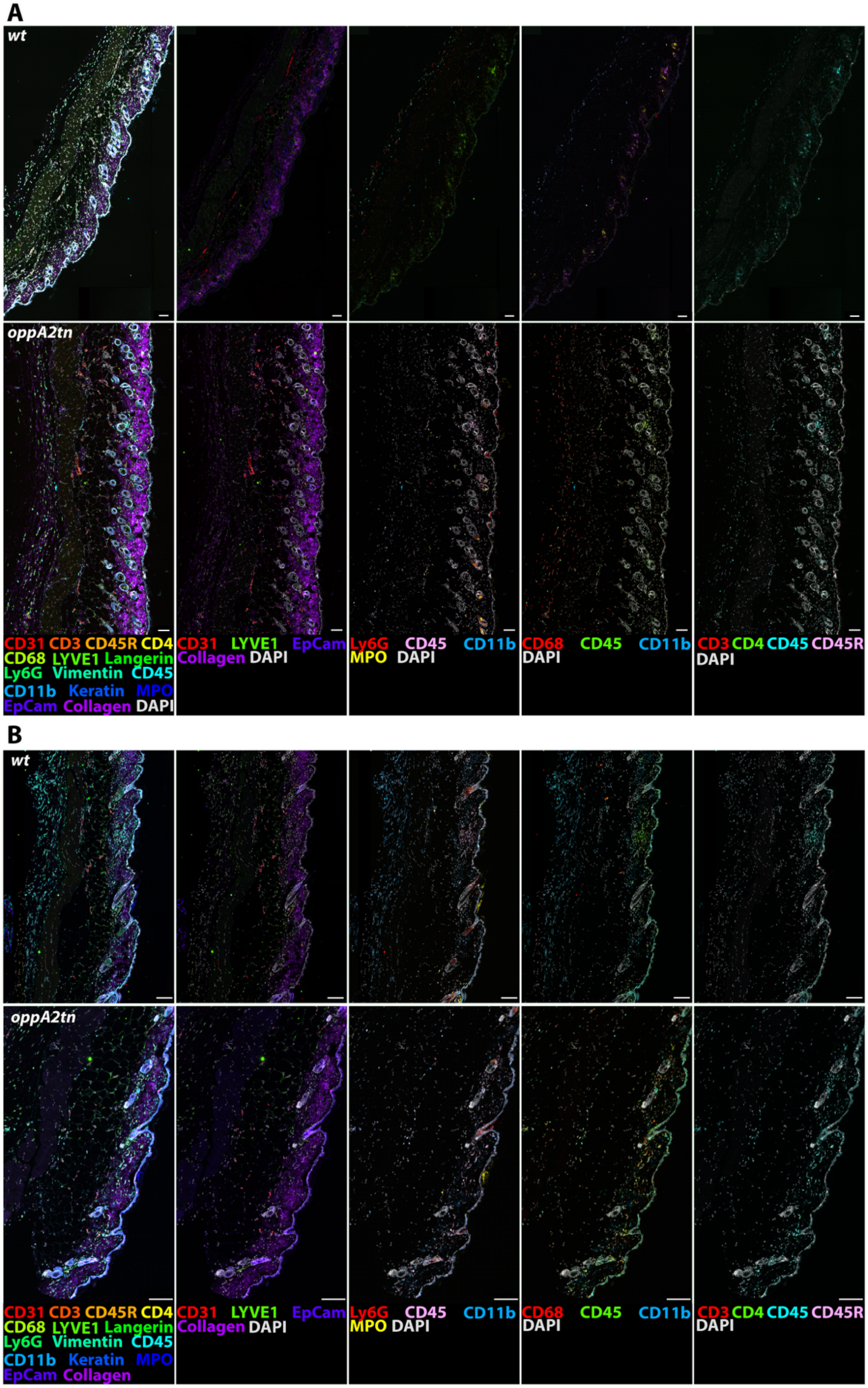
A
wt
oppA2tn
CD31 CD3 CD45R CD4
CD68 LYVE1 Langerin
Ly6G Vimentin CD45
CD11b Keratin MPO
EpCam Collagen DAPI
CD31 LYVE1 EpCam
Collagen DAPI
Ly6G CD45 CD11b
MPO DAPI
CD68 CD45 CD11b
DAPI
CD3 CD4 CD45 CD45R
DAPI
B
wt
oppA2tn
CD31 CD3 CD45R CD4
CD68 LYVE1 Langerin
Ly6G Vimentin CD45
CD11b Keratin MPO
EpCam Collagen
CD31 LYVE1 EpCam
Collagen DAPI
Ly6G CD45 CD11b
MPO DAPI
CD68 CD45 CD11b
DAPI
CD3 CD4 CD45 CD45R
DAPI

Supplementary Figure 5. MACSima Spatial Profiling of skin at 5 d and 8 w. (A-B) Representative images of *wt*- (upper row) and *oppA2tn*-infected (lower row) mouse skin at **(A)** 5 d and **(B**) 8 w.

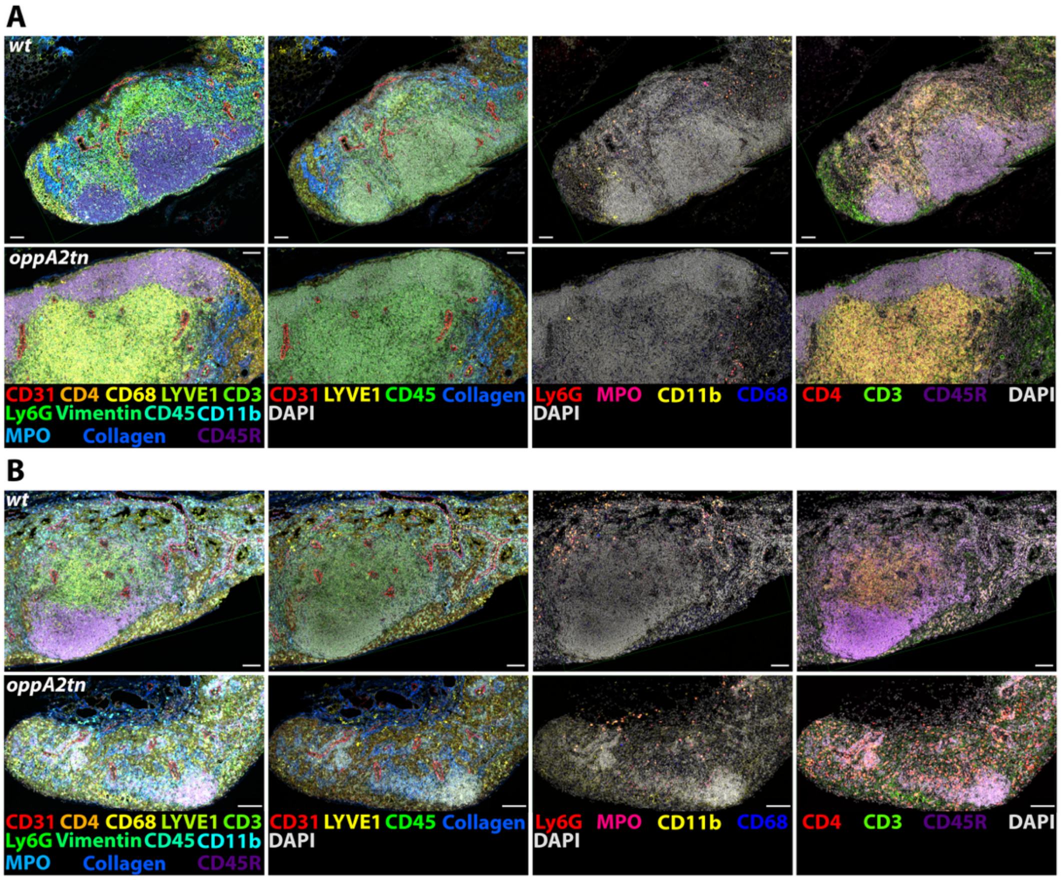

Supplementary Figure 6. MACSima Spatial Profiling of LNs at 5 d and 8 w. (A-B) Representative images of *wt*- (upper row) and *oppA2tn*-infected (lower row) mouse LNs at **(A)** 4 w and **(B**) 8 w.

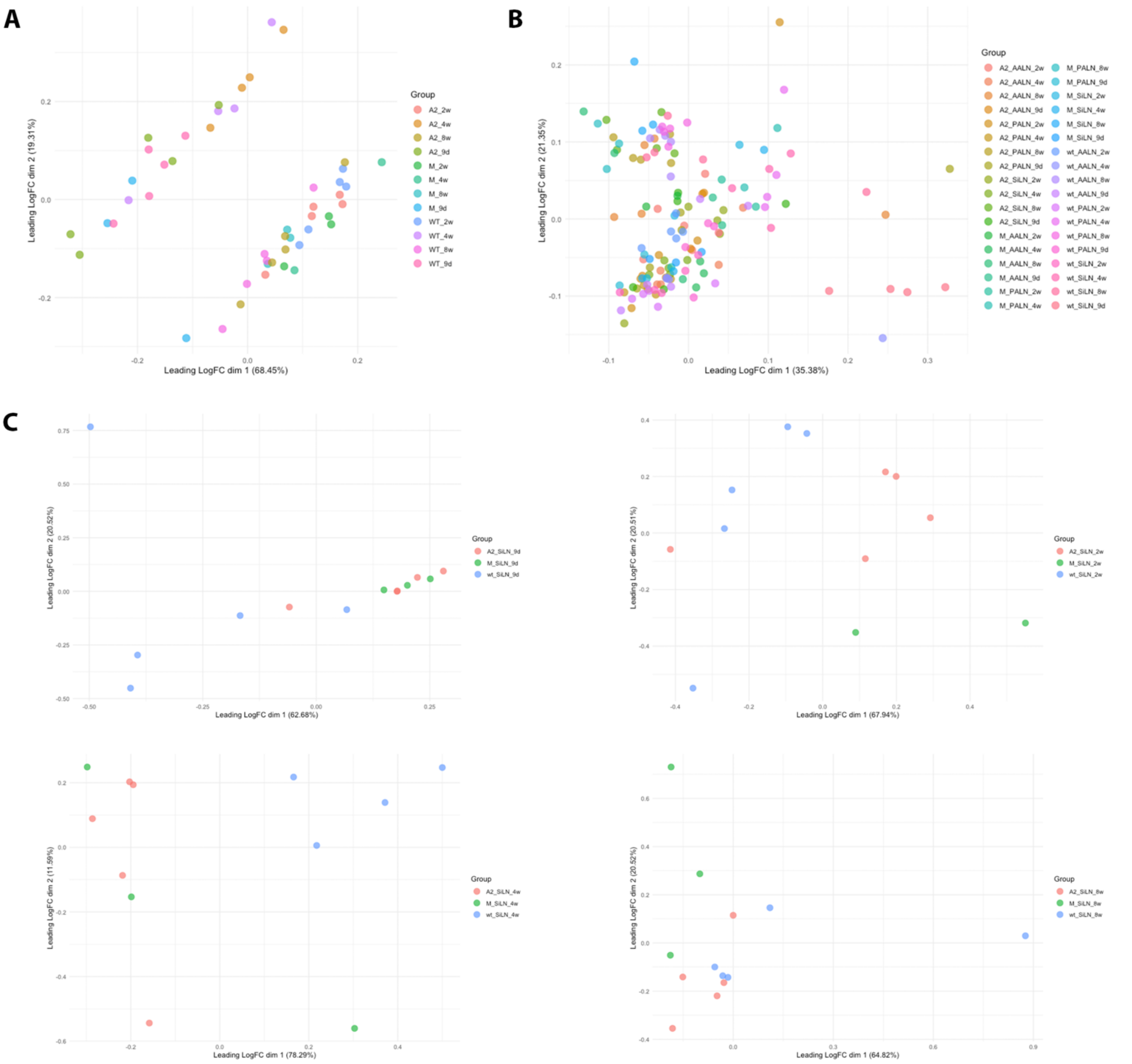

Supplementary Figure 6. PCA plots Nanostring transcriptional data for **(A)** skin at all timepoints, **(B)** all LNs at all timepoints, **(C)** SiLN for each timepoint. PCA plots were generated in R studio.

Supplementary References

1. Kawabata H, Norris SJ, Watanabe H. BBE02 Disruption Mutants of *Borrelia burgdorferi* B31 Have a Highly Transformable, Infectious Phenotype. Infection and Immunity. 2004;72(12):7147–54.

2. Groshong AM, McLain MA, Radolf JD. Host-specific functional compartmentalization within the oligopeptide transporter during the *Borrelia burgdorferi* enzootic cycle. PLOS Pathogens. 2021;17(1):e1009180.

3. Fortune DE, Lin YP, Deka RK, Groshong AM, Moore BP, Hagman KE, et al. Identification of Lysine Residues in the *Borrelia burgdorferi* DbpA Adhesin Required for Murine Infection. Infection and Immunity. 2014;82(8):3186–98.
